# Cation-Stress-Responsive Transcription Factors SltA and CrzA Regulate Morphogenetic Processes and Pathogenicity of *Colletotrichum gloeosporioides*

**DOI:** 10.1371/journal.pone.0168561

**Published:** 2016-12-28

**Authors:** Amit K. Dubey, Shiri Barad, Neta Luria, Dilip Kumar, Eduardo A. Espeso, Dov B. Prusky

**Affiliations:** 1 Department of Postharvest Science of Fresh Produce, Agricultural Research Organization, the Volcani Center, Bet Dagan, Israel; 2 Department of Plant Pathology and Microbiology, The Robert H. Smith Faculty of Agriculture, Food and Environment, The Hebrew University of Jerusalem, Rehovot, Israel; 3 Department of Molecular and Cellular Biology, Centro de Investigaciones Biológicas (C.I.B.), Madrid, Spain; Woosuk University, REPUBLIC OF KOREA

## Abstract

Growth of *Colletotrichum gloeosporioides* in the presence of cation salts NaCl and KCl inhibited fungal growth and anthracnose symptom of colonization. Previous reports indicate that adaptation of *Aspergillus nidulans* to salt- and osmotic-stress conditions revealed the role of zinc-finger transcription factors SltA and CrzA in cation homeostasis. Homologs of *A*. *nidulans* SltA and CrzA were identified in *C*. *gloeosporioides*. The *C*. *gloeosporioides* CrzA homolog is a 682-amino acid protein, which contains a C_2_H_2_ zinc finger DNA-binding domain that is highly conserved among CrzA proteins from yeast and filamentous fungi. The *C*. *gloeosporioides* SltA homolog encodes a 775-amino acid protein with strong similarity to *A*. *nidulans* SltA and *Trichoderma reesei* ACE1, and highest conservation in the three zinc-finger regions with almost no changes compared to ACE1 sequences. Knockout of *C*. *gloeosporioides crzA* (Δ*crzA*) resulted in a phenotype with inhibited growth, sporulation, germination and appressorium formation, indicating the importance of this calciu006D-activated transcription factor in regulating these morphogenetic processes. In contrast, knockout of *C*. *gloeosporioides sltA* (Δ*sltA*) mainly inhibited appressorium formation. Both mutants had reduced pathogenicity on mango and avocado fruit. Inhibition of the different morphogenetic stages in the Δ*crzA* mutant was accompanied by drastic inhibition of chitin synthase A and B and glucan synthase, which was partially restored with Ca^2+^ supplementation. Inhibition of appressorium formation in Δ*sltA* mutants was accompanied by downregulation of the MAP kinase *pmk1* and carnitine acetyl transferase (*cat1*), genes involved in appressorium formation and colonization, which was restored by Ca^2+^ supplementation. Furthermore, exposure of *C*. *gloeosporioides* Δ*crzA* or Δ*sltA* mutants to cations such as Na^+^, K^+^ and Li^+^ at concentrations that the wild type *C*. *gloeosporioides* is not affected had further adverse morphogenetic effects on *C*. *gloeosporioides* which were partially or fully restored by Ca^2+^. Overall results suggest that both genes modulating alkali cation homeostasis have significant morphogenetic effects that reduce *C*. *gloeosporioides* colonization.

## Introduction

When attacking fruit, filamentous fungi can modulate their host's pH [1]. This enables them to control pathogenicity by activating pH-regulated processes that are modulated by the transcription factors PacC and AreB in *C*. *gloeosporioides* [2–4]. Pathogens can also grow in extreme environmental niches, such as in the presence of salts, heat and drought, and in aquatic habitats, among others [5–8]. These environments can induce osmotic, heat and oxidative stress, as well as nutrient deprivation [6]. While pH modulation represents a mechanism for the regulation of biochemical and physiological processes by the fungus, it may also induce the activation of several genes that contribute to pathogenicity [9]. Accordingly, fungi have developed sophisticated mechanisms to alleviate the extracellular stress, thereby promoting growth and survival.

In the filamentous fungus *Aspergillus nidulans*, tolerance to elevated extracellular concentrations of mono- and divalent cations requires the activity of C_2_H_2_ zinc-finger transcription factors SltA and CrzA [10]. *sltA*-deletion mutants display sensitivity to high concentrations of diverse salts [11] and increased sensitivity to arginine or mutagens [6,12]. A second element of the Slt pathway, is SltB [6,12]. SltB is a putative bi-functional protein containing a pseudo-kinase and chymotrypsin-like domains that modulate proteolytic activation of both SltA and SltB proteins [6].

CrzA mediates calcium-dependent gene regulation by binding to calcineurin-dependent response elements [13]. *crzA* deletions cause decreased conidiation, poor growth and loss of virulence in *Aspergillus fumigatus* [14] by modulating the expression of chitin synthase A (*chsA*), chitin synthase B (*chsB*) and glucan synthase A (*fks1*) [15].

Both *crzA* and *sltA* in *A*. *nidulans* contribute to alleviate the extracellular stresses related to Na^+^, K^+^, Li^+^, Cs^+^ and Mg^2+^, but not Ca^2+^, along with integrity of the cell wall [10,16]. *sltA* also plays a role in fungal development and in the production of secondary metabolites [17].

The absence of data on the roles of *sltA* and *crzA* homologs from other fungal species raised the question of the sensitivity of pathogenic fungi to cation salts and the importance of SltA and CrzA in fungi such as *Colletotrichum gloeosporioides*. The present work analyzed the phenotypic responses of wild type and *sltA-* and *crzA*-knockout mutants of a fungal pathogen of fruit to cation salts. Our results show the susceptibility of *C*. *gloeosporioides* to elevated extracellular concentrations of monovalent cations and suggest a critical contribution of *crzA* and *sltA* to a wide range of morphogenetic processes. *ΔcrzA* affect mycelial growth, and inhibition of sporulation, germination and appressorium formation, which were partially restored by Ca^2+^ treatment. The *C*. *gloeosporioides* Δ*sltA* mutants mainly showed inhibition of appressorium formation, which could be restored by Ca^2+^ supplementation. Exposure of mutant strains to Na^+^, K^+^ and Li^+^ further inhibited all of the morphogenetic responses induced by the *sltA* and *crzA* mutations, indicating the importance of these transcription factors in the modulation of cation homeostasis and fungal pathogenicity of *C*. *gloeosporioides*.

## Materials and Methods

### Fungal isolates, media, growth conditions and inoculation conditions

*C*. *gloeosporioides* strain Cg-14 was obtained from a decayed avocado fruit (*Persea americana* 'Fuerte') in Israel [18] and routinely cultured on M_3_S media [19] containing (per L): 2.5 g MgSO_4_·7H_2_O (Merck), 2.7 g KH_2_PO_4_ (Merck), 1 g Bacto peptone (Becton Dickinson), 1 g Bacto yeast extract (Becton Dickinson), 10 g sucrose (Duchefa Biochemie) and 2% (w/v) agar [20]. Growth and sporulation of the *C*. *gloeosporioides* wild-type (WT) and mutant strains (developed as described in the next sub-section) in the presence of different salt solutions were assessed on salt-amended glucose minimal media (GMM) [21] after inoculation with a 5-mm diameter disc of *Colletotrichum* culture from the M_3_S media and incubation for 6 days. The GMM were prepared according to Käfer [21] with 2% agar, adjusted to pH 6.5. The media were amended with different concentrations of KCl (25–1000 mM), NaCl (25–1000 mM), LiCl (5–100 mM) or CaCl_2_ (10 mM) as indicated in each specific experiment. Growth was evaluated 6 days after inoculation and incubation at 25°C. For sporulation studies, conidia were obtained by adding 5 mL of sterile distilled water, scraping the petri dish with a Drigalski spatula and filtering through Miracloth. Conidia were visualized with a BX60F-3 microscope (Olympus America, Melville, NY, USA) and counted using a hemocytometer.

To determine germination and appressorium formation in the WT, ectopic and Δ*sltA* and Δ*crzA* mutant strains, conidia were grown in M_3_S broth for 8 days and then filtered through Miracloth, and washed by centrifugation at 11,900 X *g* for 5 min.

Germination and appressorium formation of *C*. *gloeosporioides* WT, ectopic, and Δ*sltA-* and Δ*crzA*- strains were monitored in three 5-μL drops of salt solution on glass slides as described previously [22]. A conidial suspension (10^6^ conidia/mL) of each isolate was prepared with the salt concentrations described in each specific experiment. The slides were incubated in closed petri dishes containing moistened filter paper for 12–15 h at 24°C. Conidial germination and appressorium development were monitored in three microscopic fields on each of five replicates using a 40X Olympus microscope. Conidia were considered germinated when the germination tube was at least three times longer than the conidia. Appressorium formation was considered positive when a melanized brown structure appeared at the hyphal tip of the germinated conidia. Germination and appressorium formation by the WT strain were set to 100% and these values were compared to the mutant and ectopic strains.

For fruit inoculation, freshly harvested avocado cv. Fuerte fruit from an orchard in Kibbutz Givat Brenner (Israel) and mango cv. Keith from an orchard in Moshav Ramot (Israel) were used as previously described [22]. The fruits used in the manuscript were purchased from a packing house where no specific permissions were requiered since we paid for the fruit. The owner of the packinghouse is under the name Shoam. The orchard where fruit was grown was not visited by us at all for this research. Conidia of the various *C*. *gloeosporioides* strains collected from culture growth on M_3_S were inoculated on the avocado and mango fruit peel by placing 5 μL of conidial suspension (10^6^ conidia/mL) on each of three longitudinally spaced inoculation spots per side of 10 different fruit per treatment (90 inoculation replicates per treatment). In some experiments, inoculated mango fruit discs (15 mm diameter) consisted only of peel. After inoculation, the fruit or disks were incubated for 24 h at 22°C and 95% relative humidity in covered plastic containers containing wet paper towels, and were then further incubated until symptoms were observed. The average decay diameter and statistical analysis are reported. The inoculation experiments were repeated three times and one representative experiment is presented. To determine the effect of 10 mM CaCl_2_ on the pathogenicity of *Colletotrichum* wild type, Δ*sltA-* and Δ*crzA* mutants strains, the fruit were inoculated as before using conidia diluted in a 10 mM CaCl_2_ solution followed by incubation and evaluation as described.

### *C*. *gloeosporioides* sltA/crzA gene disruption

The knockout construct was generated by PCR amplification of 530 bp of the 5′ and 580 bp of the 3′ flanking fragments of the coding region of *C*. *gloeosporioides sltA* from the full gene (NCBI, GenBank a.n. KU925876). To prepare the *crzA*-knockout construct, 624 bp of the 5′ and 694 bp of 3′ flanking fragments of the full *crzA* gene (NCBI, GenBank a.n. KX714301) coding regions were amplified. For *sltA*, primer sets attBsltA_5'F + attBsltA_5'R and attBsltA_3'F + attBsltA_3'R, and for *crzA* attBcrzA_5'F + attBcrzA_5'R and attBcrzA_3'F + attBcrzA_3'R (S1 Table) were used to amplify the 5′ and 3′ fragments, respectively. The PCR mixture included 30 ng genomic DNA, 10 pmol oligonucleotide and PCR Ready Mix (Thermo Scientific). The construct was generated through GATEWAY technology according to the manufacturer’s instructions [23]. The plasmid was isolated, sequenced, and digested with *Not*I to release the deletion construct. Electroporation of germinating conidia was performed essentially as described previously [24]. Briefly, strain Cg-14 isolates were cultured on solid M_3_S medium for 14 days. Conidia were collected in pea juice [25], adjusted to 10^6^ conidia/mL, and incubated at 28°C for 2.5 h to initiate germination. The germinated conidia were collected, washed with cold electroporation buffer (1 mM n-2-hydroxyethylpiperazine-N-2-ethenesulfonic acid and 50 mM mannitol, pH 7.5), concentrated to 10^8^ conidia/mL, and 100-μL aliquots were distributed in cold electroporation cuvettes (Bio-Rad). Electroporation and transfer to regeneration medium were performed as described previously [23]. Transformed colonies appeared 3 to 4 days after electroporation. The transformants were regrown as single-spore colonies on M_3_S agar with hygromycin B at 100 mg/L, and DNA was extracted with the Master Pure Yeast Purification Kit (Epicentre Biotechnologies). Two methods were used to confirm deletion of the *sltA* or *crzA* gene. First, specific primers were designed outside the cassette region (sltA/crzA_5’ctrl _F and sltA/crzA _3’ctrl_R). The sltA/crzA_5’ctrl _F + Hyg_5'R primers were used to check for correct insertion at the 5' locus and the hygromycin_3'F + sltA/crzA_3’ctrl_R primers were used to check for correct insertion at the 3' locus. The set of primers attbSltA_5'F + Hyg_5'R and Hyg_3'F + attbSltA_3'R and attbcrzA_5'F + Hyg_5'R and Hyg_3'F + attbcrzA_3'R was used to verify the ectopic transformants. In the case of homologous integration, the mutants showed the correct PCR fragments of approximately 750 bp from the 5' and 3' region, respectively, which were sequenced for verification; a second method was used to check the relative expression of *sltA i*n Δ*sltA10A* and Δ*sltA21C* mutants or expression of *crzA* in the Δ*crzA31A* and Δ*crzA36C* mutants, compared to the WT and ectopic *sltA1C* and *crzA37B*, confirming deletion of both genes (S3D and S3E Fig).

### Nucleic acid analysis

RNA extraction was carried out using the SV Total RNA Isolation Kit (Promega). Purity of the extracted RNA was assayed in an ND-1000 spectrophotometer (NanoDrop Technologies Inc.), and it was then stored at -80°C until further analysis.

For RNA extraction of growing hyphae, conidia of the WT, mutant and ectopic strains were inoculated at 10^6^ conidia/mL in 40 mL of primary M_3_S medium in 125-mL flasks and grown for 2–3 days at 22–24°C in a shaking incubator at 150 rpm; they were then harvested by filtration through a sterile Buchner funnel fitted with filter paper. The hyphal mat was washed twice with 40 mL sterile distilled water, and the washed mycelia were resuspended in 40 mL fresh GMM pH 6.5 for 48 h. Hyphae from the strains were recovered after 2 days and immediately transferred to liquid nitrogen, and 80- to 100-mg samples of the hyphae were lyophilized and used for RNA extraction to determine the genes' relative expressions.

For RNA extraction of germinating *C*. *gloeosporioides* producing conidia, the method of ‘pathogenic’ germination according to Barhoom and Sharon [26] was used. Samples of conidia suspensions in DDW (10 mL) of WT, Δ*sltA*, mutant and ectopic strains of *C*. *gloeosporioides* (10^6^ conidia per dish) were spread onto 20-cm diameter petri dishes and incubated for 12 to 14 h. The germinated appressorium-producing conidia were then harvested by scraping them off the petri dishes and concentrating by centrifugation at 3200*g* for 20 min. The pellet containing germinating conidia and appressoria was harvested by filtering, lyophilized, and subjected to RNA extraction and evaluation of the relative expression of MAP kinase (MAPK) *Pmk1* and carnitine acyl transferase (*cat*) [27] using the primers described in S1 Table.

The effect of *crzA* on fungal growth was determined by testing the relative expression of genes related to cell wall development [15] using the primers described in S1 Table. The mutant, WT and ectopic strains were grown on solid GMM for 5 days. The mycelium from the culture plates was scraped using sterile blades and immediately transferred to liquid nitrogen. The lyophilized mycelia were subjected to RNA extraction and relative expression determination of the markers *chsA*, *chsB* and *fsk1A* in the knockout mutants vs. WT strain.

### Gene-expression analysis by qRT-PCR

To examine gene expression, RNA was extracted and 1 μg of total RNA was reverse-transcribed with the Reverse-it First-Strand Synthesis Kit (ABgene) according to the manufacturer's protocol. cDNA samples were diluted 1:10 (v/v) with ultrapure water. Real-time qPCR was performed with the StepOnePlus System (AB, Applied Biosystems). PCR amplification was performed with 3.4 μL of cDNA (about 340 ng of cDNA) template in 10 μL of a reaction mixture containing 6.6 μL mix from the SYBR Green Amplification kit (ABgene) and 300 nM primers. S1 Table lists the forward and reverse primers for each of the indicated genes. PCR was carried out with the following cycling program: 10 min at 94°C, followed by 40 cycles of 94°C for 10 s, 60°C for 15 s, and 72°C for 20 s. The samples were subjected to melting-curve analysis, with efficiencies close to 100% for all primer pairs, and all products showed the expected size of 70 to 100 bp. All of the samples were normalized to *18S* rRNA expression levels and the values were expressed as the change (increase or decrease) in level relative to the WT sample. Results were analyzed with StepOnePlus v.2.2.2 software. Relative quantification was performed by the ΔΔC_T_ method [28]. The ΔC_T_ value was determined by subtracting the C_T_ results for the target gene from those for the endogenous control gene and normalized against the calibration sample to generate the ΔΔC_T_ values. Each experiment was performed in triplicate, and three different biological experiments were conducted. One representative set of results is presented as mean values of 2^-ΔΔCT^ ± SE for each treatment.

### Staining with CFW and fluorescence microscopy

Staining was performed according to the protocol of Hageage and Harrington (2003) available at http://www.mycology.adelaide.edu.au/) with some modifications [29]. Calcofluor white at 0.1% (w/v) solution and 10% KOH were prepared in sterile distilled water. One drop of each solution was mixed on the center of clean slide. Specimen was placed in the solution and cover with coverslip. Excess fluid was blot dried with tissue paper. Specimen was observed using the OLYMPUS IX 81 (Japan) inverted laser scanning confocal microscope (FLUOVIEW 500) equipped with a 405 nm diode laser and 60X1.0 NA PlanApo water immersion objective was excited by 405 nm light and the emission was collected through an BA 430–460 filter.

### Data analysis

Statistical differences between results of the RT-qPCR analysis were analyzed by Student's t-test, and growth, sporulation, germination, appressorium formation and colonized area at a single time point were analyzed with Tukey-Kramer multiple comparison test at 0.95 confidence interval. Analysis was conducted by JMP software (SAS Institute). Significance was indicated (for growth, sporulation, germination and appressoria formation) by either lower or upper case or (for relative gene expression) by asterisks (*).

## Results

### Effect of cation salts on growth and colonization of *C*. *gloeosporioides*

**Fig 1:**
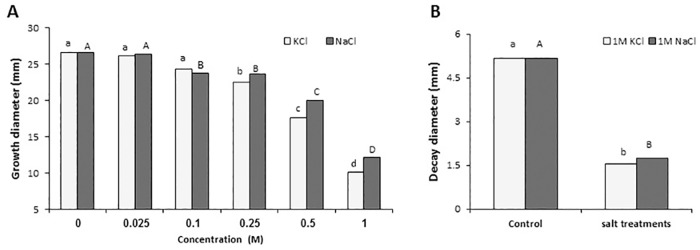
Effect of NaCl and KCl cation salts on growth of *C*. *gloeosporioides* in solid SM media (A) and colonization of mango fruit slices following dip treatment 24 hours after inoculation (B). Fruit discs were inoculated with 10 μL conidial suspension and incubated at 24°C under high humidity. Evaluation fungal growth in plates and colonization of discs was carried out 3 days after inoculation. Average of 6 inoculated discs of a single biological experiment out of duplicate experiments is presented. Columns with different letters (lower or upper case) are significantly different at P≤0.05 according to Tukey-Kramer multiple comparison test.

Growth of *C*. *gloeosporioides* in solid SM media amended with increasing concentrations of NaCl and KCl staring from 25 and up to 1000 mM inhibited fungal growth by 54.3% and 61.9% respectively (Fig 1A). The inhibitory effect of cations on fungal growth was tested also for the inhibition of colonization of *Colletotrichum* on mango fruit discs. Inoculation of mango cv. Shelly discs followed by dip treatment of the disc in 1.0 M NaCl and KCl,12 and 24 h after infection, showed that fully inhibited anthracnose symptoms caused by *C*. *gloeosporioides* when analyzed 5 days later (Fig 1B). These results lead us to the study of the differential susceptibility of C. gloeosporioides to cations salts.

### Identification of genes encoding SltA and CrzA homologs in *C*. *gloeosporioides*

BlastP search in the *C*. *gloeosporioides* Cg-14 database against the SltA amino acid sequence (AN2919) confirmed the presence of a homolog of *A*. *nidulans sltA*, entry CGLO_02275, with 775 amino acids. BlastP search in the *C*. *gloeosporioides* Nara gc5 protein database also identified entry XP_007278073.1, previously annotated as a protein similar to the factor ACE1 from *Trichoderma reesei*. Multiple sequence alignment using SltA sequences from *A*. *nidulans* and *C*. *gloeosporioides* and the *T*. *reesei* ACE1 sequence revealed discrepancies in the automatic prediction for the SltA ortholog of *C*. *gloeosporioides* strain gc5 (S1 Fig). Thus, the 5'-AGGCA sequence is probably a common core sequence for all SltA/ACE1 homologs, as shown for *A*. *nidulans* SltA [5].

The CrzA protein sequence from *A*. *nidulans* (BAE_94327.1) was used to search for homologs in the *C*. *gloeosporioides* Cg-14 protein database using BlastP, and the results showed highest match (E-value = 1e^-^124) with a hypothetical protein (CGLO_04525) and sequence ID EQB55543.1. Multiple sequence alignment of CrzA protein sequences from: *A*. *nidulans* BAE94327, *A*. *fumigatu*s EAL88401, *Neurospora crassa* EAA32849, *Magnaporthe oryzae* XP_359644.1, *Chaetomium globosum* EAQ88414, *Coccidioides immitis* EAS33001, and *Phaeosphaeria nodorum* EAT87393 showed that the sequences contain three zinc-finger domains. Of these (S2A Fig), two zinc fingers (Znf1 and Znf2) were canonical C_2_H_2_, whereas the third (Znf3) had an unusual Cys_2_HisCys structure [10]. The sequence similarities also showed the presence of two possible overlapping calcineurin-docking domains (CDDs) (S2B Fig), of which CDD2 has been shown to be necessary for binding of calcineurin to CrzA of *A*. *nidulans* [30]. The presence of conserved motifs in the C-terminal region with respect to the zinc-finger domain indicated the existence of possible common regulatory domains and functions for all members of this family of transcription factors.

### Development of *C*. *gloeosporioides* *ΔsltA/ΔcrzA* mutant strains and their phenotypic analysis

To assess the function of *C*. *gloeosporioides sltA/crzA*, gene deletions were carried out to generate Δ*sltA/*Δ*crzA* mutant strains by homologous recombination (S3 Fig), replacing the intact *sltA/crzA* with the sltA5'-Hyg-sltA3' and crzA5'-Hyg-crzA3' deletion cassette, respectively [23]. A double-crossover homologous recombination event resulting in replacement of the original *C*. *gloeosporioides sltA/crzA* sequence with the sltA5'-Hyg-sltA3' or crzA5'-Hyg-crzA3' cassettes was performed as described in Materials and Methods (S3A–S3C Fig). The pairs of primers used to create the construct were attBsltA/crzA_5'F—attBsltA/crzA_5’R for the 5' end and attBsltA/crzAAA_3'F—attBsltA/crzAAA_3'R for the 3' end (S1 Table). PCR analyses of the WT strain, ectopic colony, and independent *sltA/crzA*-disrupted colonies are shown in S3B and S3C Fig. *sltA/crzA*_5’ctrl_F, flanking a position upstream of the *sltA*/*crzA*:*HYG3* region, and reverse primer Hyg_5'R, located on the hygromycin cassette, were used to identify positive *C*. *gloeosporioides sltA /crzA* gene replacement at the 5′ locus. Hyg_3'F from the hygromycin cassette and sltA/crzA_3’ctrl_R flanking the *crzA*:*HYG3* region were used to identify *sltA/crzA* gene replacement at the 3′ locus. *sltA/crzA* attB primers for the 5' and 3' ends were used for the WT as a control. attBsltA/crzA_5'F—Hyg_5'R primers were used as a positive control for the ectopic strains, to confirm random integration of the 5'-sltA/crzA:HYG3 cassette. Hyg_3'F—attBsltA/crzA_3'R primers were used as a positive control for the ectopic strains, to confirm random integration of the 3'-sltA/crzA:HYG3 cassette. Conidia of WT, mutant and ectopic strains were inoculated at 10^6^ spore/mL in 40 mL of primary M_3_S medium in 125-mL flasks and grown for 2–3 days at 22–24°C in a shaking incubator at 150 rpm; they were then harvested by filtration through a sterile Buchner funnel fitted with filter paper. The hyphal mat was washed twice with 40 mL of sterile distilled water, the washed mycelia were resuspended in 40 mL of fresh GMM pH 6.5 for 48 h, and the relative gene expression was analyzed. No expression of *sltA* in Δ*sltA10A* or Δ*sltA21C* mutants and no expression of *crzA* in the Δ*crzA31A* and Δ*crzA36C* mutants were detected when comparing the WT and ectopic *sltA1C* and *crzA37B*, confirming deletion of both genes (S3D and S3E Fig).

**Fig 2:**
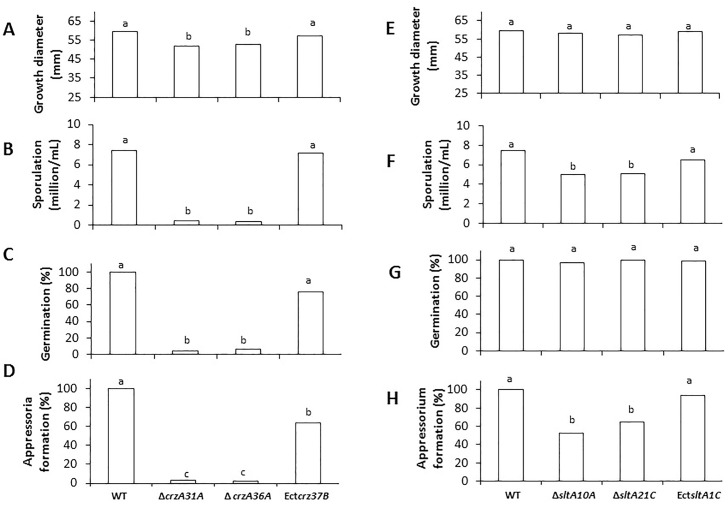
Growth, sporulation, germination and appressorium formation of wild-type (WT), *ΔcrzA* (*ΔcrzA31A*, *ΔcrzA36A*), Ect*crzA37B* (ectopic), *Δslt*A (*ΔsltA10A* and *ΔsltA21C*) and Ect*sltA1C C*. *gloeosporioides* strains in amended glucose minimal media. Strains were disc-inoculated on glucose minimal media and incubated for 5 days at 25°C. (A, E) Radial colony growth, (B, F) sporulation, (C, G) germination and (D, H) appressorium formation were evaluated after 16 h of incubation on glass slides at 25°C. Experiments were repeated three times and results of a single representative experiment are shown. Columns with different letters are significantly different at P ≤ 0.05 according to the Tukey-Kramer multiple comparison test.

**Fig 3:**
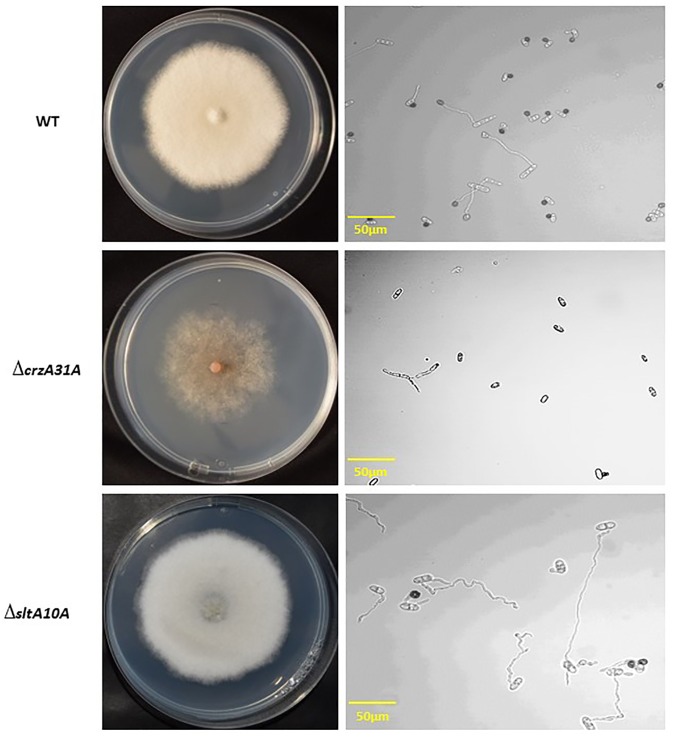
Phenotypic analysis of radial colony growth (A) and appressorium formation (B) by wild-type (WT), *ΔcrzA31A* and *ΔsltA10A* strains of *C*. *gloeosporioides*. Cultures were point-inoculated with an agar disc on agar plates containing glucose minimal media at pH 6.5. Pictures of germinated spores were taken from respective cultures 6 days later at 25°C and germinated from a spore suspension 24 h later.

Growth of *ΔcrzA* strains on GMM was inhibited by 12–15% as compared to the WT (59.6 ± 2.5 mm diameter compared to 51.6 ± 2.5 mm), but the mycelial density was considerably reduced compared to the WT and ectopic strains after 6 days at 28°C (Figs 2A and 3). Furthermore, sporulation and germination capabilities overnight at high relative humidity on water over glass slides, as well as appressorium formation, were inhibited by more than 95–98% in the *ΔcrzA* strain compared to the WT and ectopic strains, while there was a small difference in appressoria formation between the WT and the ectopic strain (Figs 2B–2D and 3).

In contrast, growth of WT, ectopic and *ΔsltA* strains was similar (59.6 ± 2.5 mm diameter) after 6 days at 28°C, and germination capabilities were almost 100%, with no differential effect on germination (Figs 2E, 2G and 3). However, analysis of sporulation capabilities of the *ΔsltA* strains showed a reduction of spore yield from 7.42 x 10^6^ spore/mL in the WT to 5 x 10^6^ spore/mL (Fig 2F), and 36–47% inhibition of appressorium formation in germinated spores compared to the WT and ectopic strains (Fig 2H).

These results indicate that while *crzA* has a drastic morphogenesis-modulating effect on growth, sporulation, germination and appressorium formation, the *sltA* mutants partially modulate sporulation but significantly modulate appressorium formation (Figs 2F–2H and 3).

Interestingly, growth of the mutant under stress conditions affected the color changes in the plate that could indicate the presence of secondary metabolites. While no identification of secondary metabolites were reported in *C*. *gloeosporioides* of tropical fruits (avocado and mango), the induced cation stress may contribute to this color changes as reported for *C*. *gloeosporioides* in yam [31].

### Effect of *ΔsltA* and *ΔcrzA* strains on colonization by *C*. *gloeosporioides*

**Fig 4:**
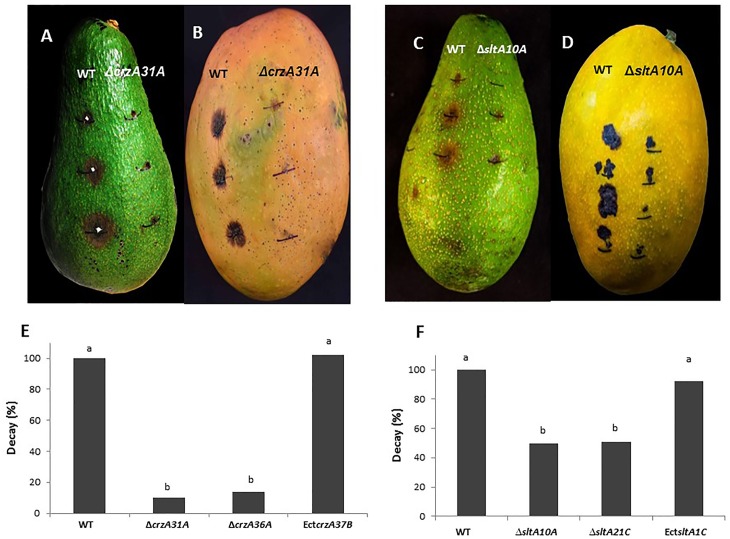
Colonization of avocado cv. Fuerte and mango cv. Shelly fruit by *ΔcrzA* (A, B) and Δ*sltA* (C, D) mutants, respectively. Colonization of avocado fruit by Δ*crzA31A* (E) and Δ*slt10A* (F) compared to wild type (WT) (100% colonization). Fresh fruit were inoculated with 10 μL conidial suspension and incubated at 24°C under high humidity for 10–12 days. Fruit with more than 0.5-cm diameter colonized tissue were considered colonized. Experiments were repeated three times and results of a single representative experiment are shown. Columns with different letters are significantly different at P ≤ 0.05 according to the Tukey-Kramer multiple comparison test.

To functionally analyze the contribution of the *crzA* and *sltA* transcription factors on fruit colonization, 5-μL drops (10^6^ conidia/mL) of WT and mutant strains were placed on the peel of avocado and mango fruit, incubated at 24°C under high humidity, and evaluated 10–12 days later. No symptom development was observed after Δ*crzA* mutant inoculation on either avocado or mango fruit (Fig 4A, 4B and 4E). While the *sltA* mutant showed partial inhibition of colonization development in avocado and mango tissue (Fig 4C, 4D and 4F), total inhibition of colonization was observed by inoculation with the Δ*crzA* mutants, suggesting that strong regulation of morphogenetic development induced by the *crzA* mutation also modulates *Colletotrichum* colonization.

### Growth, sporulation, germination and appressorium formation of *ΔsltA* and *ΔcrzA* strains on cation-amended cultures

**Fig 5:**
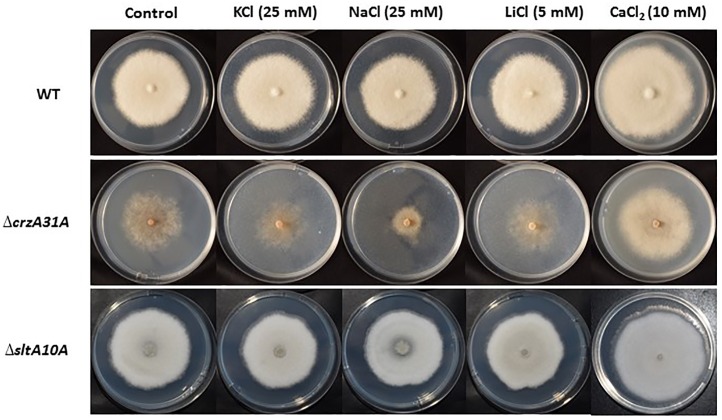
Phenotypic analysis of wild-type (WT), *ΔcrzA* and Δ*sltA* strains of *C*. *gloeosporioides* in the presence of KCl, NaCl, LiCl and CaCl_2_. Cultures were center-inoculated on agar plates containing glucose minimal media at pH 6.5 supplemented with different salt concentrations as indicated. Pictures of fungal development were taken after 5 days of incubation at 25°C.

**Fig 6:**
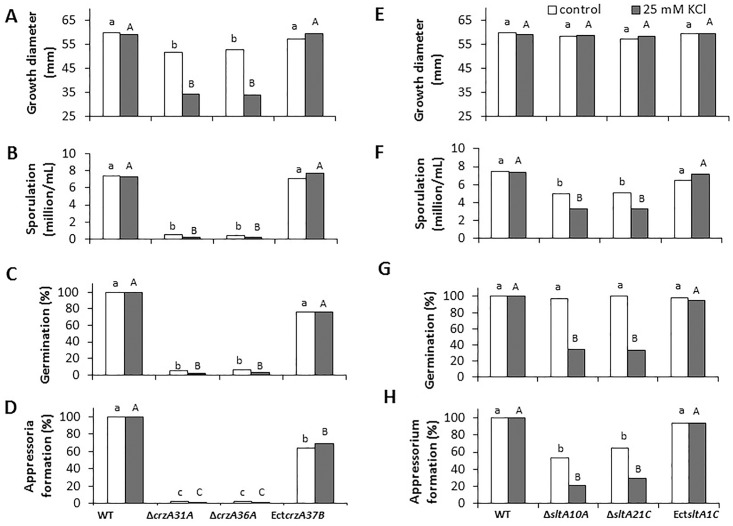
Growth, sporulation, germination and appressorium formation by the wild-type (WT), ectopic (Ect) and mutant strains of *ΔcrzA* (Δ*crzA31A*, Δ*crzA36A* and Ect*crzA37B*) and *Δslt*A (Δ*sltA10A*, Δ*sltA21C* and Ect*sltA1C*) in glucose minimal media amended (□), or not (■), with KCl. These *C*. *gloeosporioides* strains were disc-inoculated on glucose minimal media amended with 25 mM KCl and incubated for 5 days at 24°C. (A, E) Radial colony growth, (B, F) sporulation, (C, G) germination and (D, H) appressorium formation were evaluated after 16 h of incubation on glass slides at 24°C. Experiments were repeated three times and results of a single representative experiment are shown. Columns with different letters (lower or upper case) are significantly different at P ≤ 0.05 according to the Tukey-Kramer multiple comparison test.

Growth of *C*. *gloeosporioides* strains in the presence of 400 mM KCl had no effect on the growth diameter of colonies from the WT but inhibited sporulation of the WT by 63% (results not shown). To determine the differential susceptibility of *ΔsltA* and *ΔcrzA* mutants to KCl, we used the highest concentration (25 mM KCl) that had no physiological effect on growth, sporulation or germination in the WT strain (Fig 5). At this KCl concentration, growth of *ΔcrzA* mutants was inhibited by 44% compared to the WT control (Fig 6A). Sporulation, germination and appressorium formation of the *ΔcrzA* mutants were all inhibited by almost 95% without addition of KCl, and were further inhibited by the 25 mM KCl-amended media (Fig 6B–6D). For the *ΔsltA* strains, 25 mM KCl did not inhibit growth compared to the WT, but reduced sporulation by 55% compared to the WT control (Fig 6F) and inhibited germination and appressorium formation of the Δ*sltA* strains by 66 and 72%, respectively (Fig 6G and 6H). Growth of the Δ*sltA* mutant in the presence of 1 M sorbitol and KCl did not affect fungal growth (result not shown).

Growth of the *C*. *gloeosporioides* WT with 400 mM NaCl was inhibited compared to growth of the WT without NaCl. Reduction of NaCl concentration from 400 mM to 25 mM at pH 6.5 eliminated inhibition of growth in the WT and Δ*sltA* strains, but still inhibited the growth of *ΔcrzA* mutants by 50% (S4A Fig). However, at 25 mM NaCl, sporulation, germination and appressorium formation of Δ*sltA* mutants were inhibited by 32%, 32–37% and 68–75%, respectively (S4F–S4H Fig). Sporulation, germination and appressorium formation of the Δ*crzA* mutants were almost fully inhibited without NaCl, but a further increase in inhibition was observed when the mutants were grown in the presence of 25 mM NaCl-amended media (S4B–S4D Fig).

Growth of *C*. *gloeosporioides* strains in the presence of 100 mM LiCl at pH 6.5 did not affect growth or development of the WT but fully inhibited its sporulation. A decrease in LiCl concentration from 100 to 5 mM led to similar growth of both WT and Δ*sltA* strains, but the Δ*crzA* mutants still showed 40% growth inhibition (S5A Fig) with considerably reduced mat density (Fig 5). At 5 mM LiCl, the Δ*sltA* strains showed about 50% inhibition of sporulation, 70–90% inhibition of spore germination and up to 93% inhibition of appressorium formation (Fig 6F–6H). Sporulation, germination and appressorium formation of the Δ*crzA* mutants were all strongly inhibited with no LiCl, and were further inhibited by the 5 mM LiCl amendment (S5B–S5D Fig). These results suggest differential roles of CrzA and SltA in the modulation of cation toxicity in *C*. *gloeosporioides*. The *crzA* mutation by itself showed drastic morphogenetic effects of about 95% inhibition in sporulation, germination and appressorium formation, and the addition of toxic cations had a further minor enhancement of its toxic effects. However, in the Δ*sltA* strains, mainly appressorium formation was inhibited and the presence of cations further increased this inhibition, up to 90%.

### Restoration of appressorium formation and growth in strains *ΔsltA* and *ΔcrzA* by CaCl_2_

**Fig 7:**
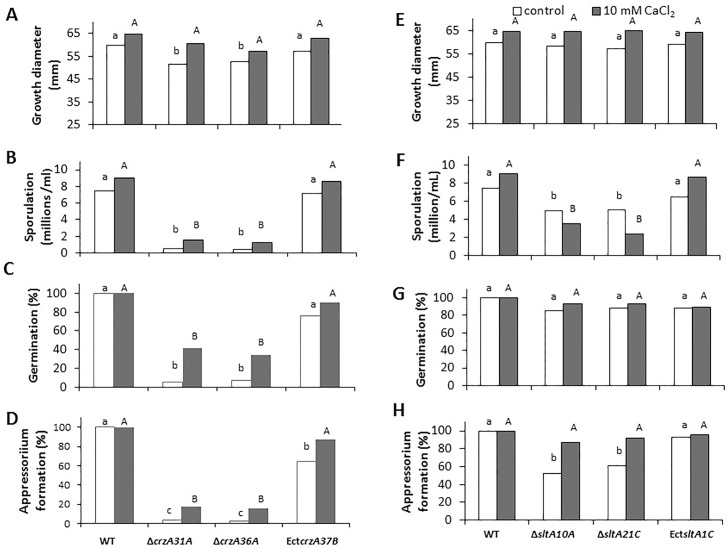
Growth, sporulation, germination and appressorium formation by the wild-type (WT), ectopic (Ect) and mutant strains of *ΔcrzA* (Δ*crzA31A*, Δ*crzA36A* and Ect*crzA37B*) and Δ*sltA* (*ΔsltA10A*, *ΔsltA21C* and Ect*sltA1C*) in glucose minimal media amended (□), or not (■), with 10 mM CaCl_2_These *C*. *gloeosporioides* strains were disc-inoculated on glucose minimal media amended with 10 mM CaCl_2_ and incubated for 5 days at 24°C. (A, E) Radial colony growth, (B, F) sporulation, (C, G) germination and (D, H) appressorium formation were evaluated after 16 h of incubation on glass slides at 24°C. Experiments were repeated three times and results of a single representative experiment are shown. Columns with different letters (lower or upper case) are significantly different at P ≤ 0.05 according to the Tukey-Kramer multiple comparison test.

**Fig 8:**
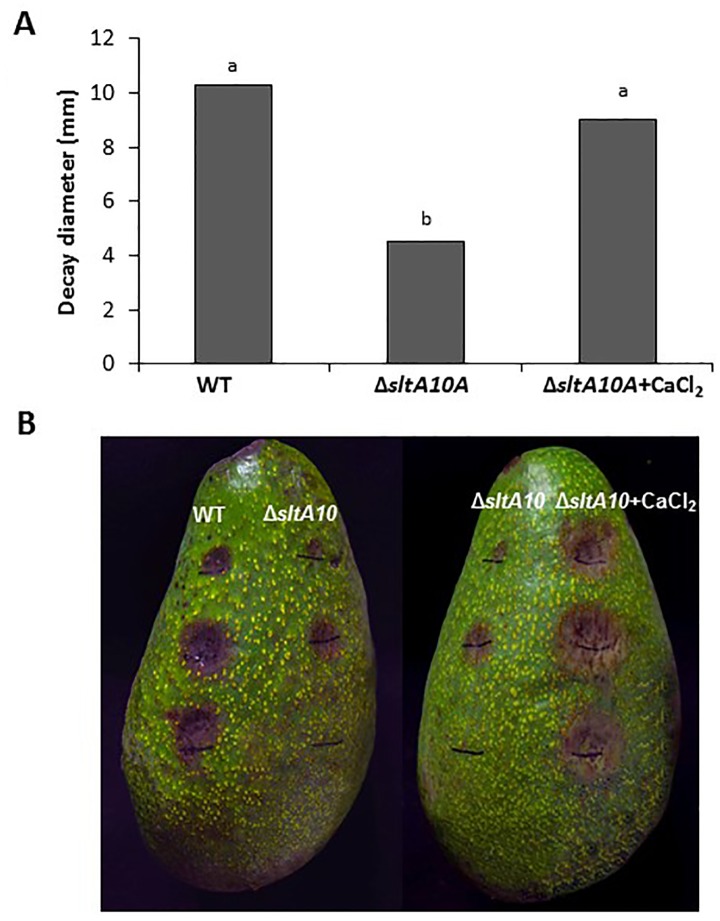
Colonization (decay diameter) of the wild-type (WT) and *ΔsltA10A* strains on avocado fruit in the presence, or not, of CaCl_2_. Decay diameter of the *ΔsltA* strains on freshly harvested avocado fruit cv. Fuerte was assessed after inoculation with 5 μL of conidial suspension (10^6^ conidia/mL) with or without 10 mM CaCl_2_ incubated under high humidity at 24°C. Decay diameter was measured 10–14 days later. Experiments were repeated three times and results of a single representative experiment are shown. Columns with different letters are significantly different at P ≤ 0.05 according to the Tukey-Kramer multiple comparison test.

Previous reports have indicated that CaCl_2_ contributes to the reduction of *Aspergillus* *ΔsltA*'s susceptibility to salt [5]. In *C*. *gloeosporioides*, growth of the WT, *crzA* mutants and ectopic strains in the presence of 10 mM CaCl_2_ was enhanced by 10%. CaCl_2_ amendment showed a significant (two- to threefold) but minor increasing effect on sporulation, germination and appressorium formation of the *ΔcrzA* mutant, but this effect was far from restoring the WT phenotype (Fig 7B–7D). This is in contrast to the *A*. *nidulans* system, where the *crzA*-null mutant is highly sensitive to similar Ca^2+^ concentrations [10,30]. However, the most striking CaCl_2_ effect in the *ΔsltA* mutants was restoration of this strain's ability to form appressoria, from 57% to 85%, similar to that found in the WT strain (Fig 7H). Similar results were found when the experiments were carried out on the peel of mango fruits, where appressorium formation by the *ΔsltA* strain, ranging between 37 and 48%, increased to 85%, similar to WT levels, when the mutant was inoculated in the presence of calcium (*ΔsltA10A* + CaCl_2_) (Fig 8, S6 Fig). This increase in appressorium formation occurred together with an increase in colonization of the *ΔsltA* strains to levels similar to those of the WT (Fig 7H). CaCl_2_ also enhanced the percentage of appressorium formation by almost fivefold in the Δ*crzA* mutant, but the level of appressorium formation was still only 17% (Fig 7D) and the colonization pattern showed no improvement compared to the WT (results not shown).

### Effect of Δ*sltA* and Δ*crzA* on gene expression modulating the morphology of *C*. *gloeosporioides* development

**Fig 9:**
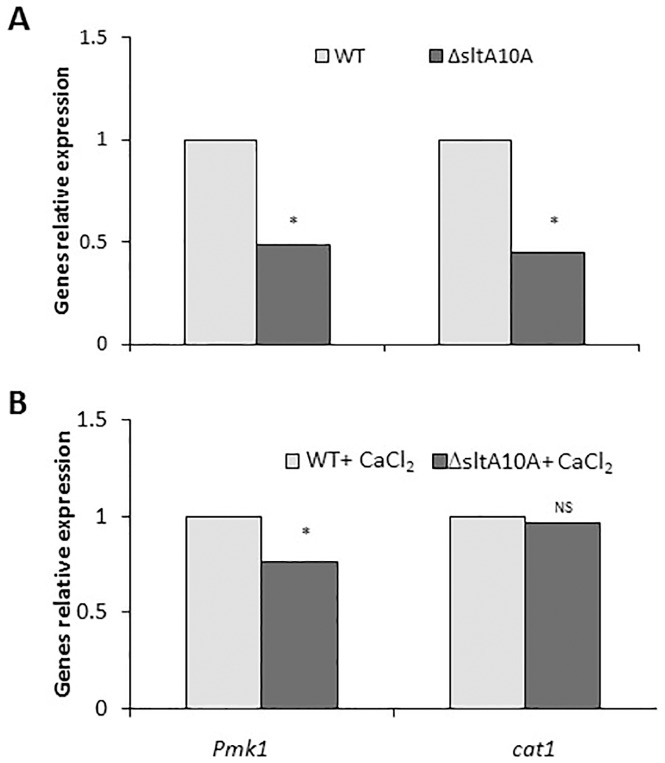
Relative expression of *pmk1* and *cat1* of wild-type (WT) and *ΔsltA* strains of *C*. *gloeosporioides* in germinating spores. RT-PCR values were normalized against *18S* rRNA and compared to the lowest expression value in the respective treatments, which was set to 1. Average ± SE of three technical replications of one single biological experiment out of three repeated experiments is presented. Experiments were repeated three times and results of a single representative experiment are shown. Average of three technical replications is presented and asterisks marked columns are significantly different at P ≤ 0.05 according to the Student’s t-test. NS represents non-significant difference.

**Fig 10:**
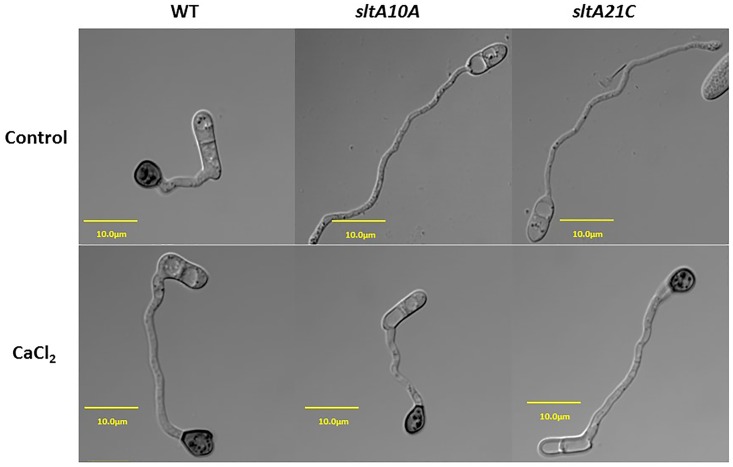
Germination and appressorium formation by spores of the *C*. *gloeosporioides* wild-type (WT), *ΔsltA10A* and *ΔsltA21C* strains in the presence of water or 10 mM CaCl_2_. Five drops of spores of the different strains were placed on a single glass slide and incubated in a humid petri dish at 24°C. Microscopic evaluation of germination and appressorium formation was evaluated 20 h later. a-appressorium, c-conidia, g-germinating tube.

To determine the possible factors modulating appressorium formation in the *ΔsltA* mutant, we analyzed the expression of two genes reported to be related with appressorium formation and melanization: *pmk1*, a MAPK involved in global regulation of appressorium formation, and *cat1*, required for host invasion during appressorium formation in *Magnaporthe* [27]. The relative expression of *pmk1* and *cat1* was inhibited by almost 50% in the *ΔsltA* mutants (Fig 9A). Addition of CaCl_2_ suppressed the lack of appressoria formation in germinating conidia of null *sltA* strains. This was in agreement with an increase of expression levels of *pmk1* and *cat1*, suggesting the importance of the *sltA* mutation in the regulation of both genes (Figs 9B and 10). Promoter analysis of *pmk1* and *cat1* in the *C*. *gloeosporioides* Cg-14 genome showed multiple 5'-AGGCA-3' sequences which are a consensus binding site for SltA and ACE1 proteins, suggesting a direct role of SltA in regulating the expression of these MAPK and *cat1* genes.

**Fig 11:**
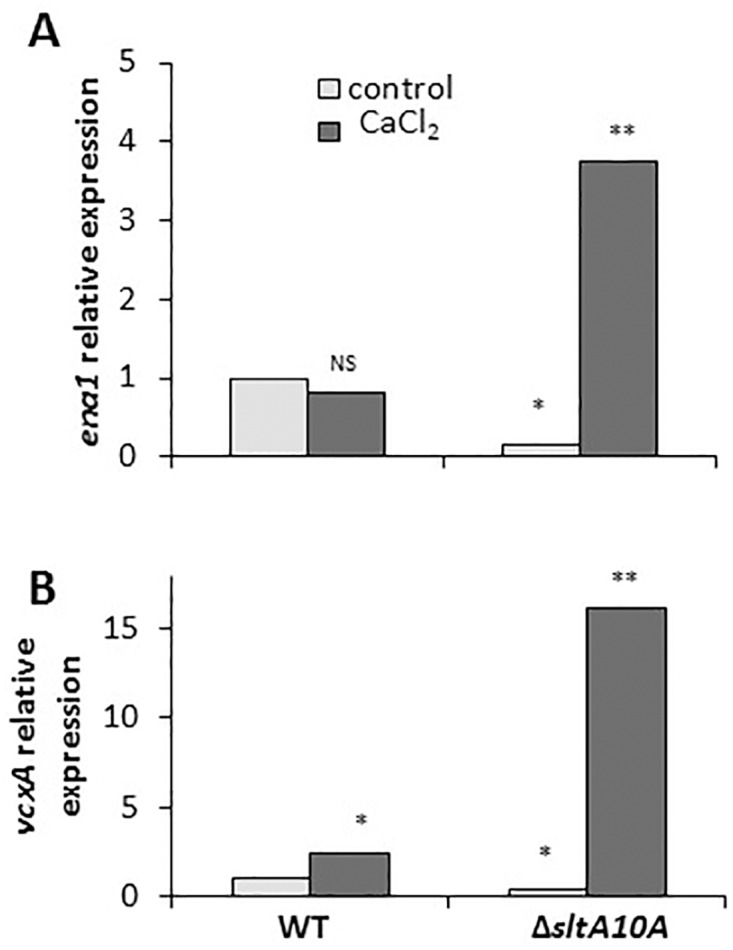
Relative expression of *ena1* (A) and *vcxA* (B) of wild-type (WT) and *ΔsltA* strains of *C*. *gloeosporioides*. RT-PCR values were normalized against *18S* rRNA and compared to the lowest expression value in the respective treatments, which was set to 1. Experiments were repeated three times and results of a single representative experiment are shown. Average of three technical replications is presented and asterisks marked columns are significantly different at P ≤ 0.05 according to the Student’s t-test. One asterisk represents lower expression and two asterisks represent higher expression. NS represents non-significant difference.

To understand the mechanism of Ca^2+^ reactivation of genes in the *ΔsltA* background, we hypothesized that in the presence of Ca^2+^, *vcxA* (a putative vacuolar Ca^2+^/H^+^ exchanger) and *ena*1 (a putative sodium/lithium exporter) are induced and consequently detoxify Ca^2+^ toxicity [10] (Fig 11). In *A*. *nidulans*, transcript levels of the putative vacuolar Ca^2+^/H^+^ exchanger gene, *vcxA*, in response to high Ca^2+^ levels and suppressor mutations of calcium-auxotrophic phenotype found that K^+^ and Na^+^ transporters provide a possible explanation for the lack of Ca^2+^ toxicity in Δ*sltA* strains [10,32]. The *C*. *gloeosporioides* Δ*sltA* strains showed an increase in the relative expression of *vcxA* and *ena*1 in *C*. *gloeosporioides* in response to Ca^2+^ (Fig 11), putatively allowing greater vacuolar storage of Ca^2+^ and a subsequent cytoplasmic detoxification of excess calcium.

**Fig 12:**
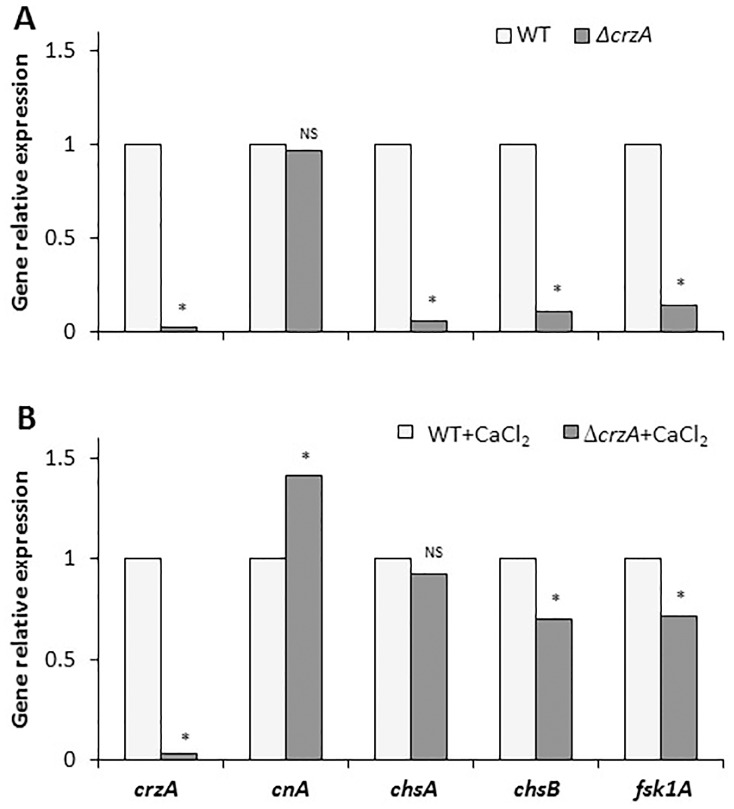
Relative expression of *crzA*, *cnA*, *chsA*, *chsB* and *fsk1* in the wild-type (WT) and *ΔcrzA* strains of *C*. *gloeosporioides* in the presence (B), or not (A), of 10 mM CaCl_2_RT-PCR values were normalized against *18S* rRNA and compared to the lowest expression value in the respective treatments, which was set to 1. Experiments were repeated three times and results of a single representative experiment are shown. Average of three technical replications is presented and asterisks marked columns are significantly different at P ≤ 0.05 according to the Student’s t-test. NS represents non-significant difference.

The Δ*crzA* mutants also showed improved growth and sporulation, germination and appressorium formation in the presence of CaCl_2_ (Fig 7A–7D), but the effect was minor compared to the significant fungal response of the *crzA* mutation (Fig 7B–7D). Stathopoulos-Gerontides *et al*. [33] reported that in *S*. *cerevisiae* and *C*. *albicans*, calcineurin becomes active when it interacts with calcium-bound calmodulin (CaM) and when the regulatory subunit is bound to calcium. Active calcineurin—CaM complex may promote dephosphorylation of CrzA and consequently induce its translocation from the cytoplasm to the nucleus to activate the transcription of genes related to cell-wall biosynthesis, such as *chsA*, *chs B* and *fksA* [15] (Fig 12). These cell wall biosynthesis-related genes were positively affected in the *C*. *gloeosporioides* *ΔcrzA* mutants that showed strong downregulation of *chsA*, *chsB* and *fsk1A* confirmed by the staining with calcofluor white where most of the mutant hyphae showed reduced staining compared to the WT (S7 Fig). The relative expression of these genes was restored by CaCl_2_ in *in-vitro* experiments (Fig 12), but no restoration of pathogenicity was observed during fruit colonization (results not shown).

## Discussion

### The transcription factors SltA and CrzA in *C*. *gloeosporioides*

The sensitivity of growth and colonization of fruits by *C*. *gloeosporioides* when exposed to cation salts as NaCl and KCl, suggested that sophisticated mechanisms to alleviate the extracellular stress promoting growth and survival may present in plant pathogens, as described previously in *Aspergillus*.

The transcription factors SltA and CrzA are important for cation adaptation and homeostasis in *Aspergillus* [5,10]. Although SltA and CrzA homologs have been found in several filamentous fungi, this is the first reported functional analysis of *sltA* and *crzA* in the plant-pathogenic fungus *C*. *gloeosporioides*. SltA homologs were found mostly in fungi belonging to the subphylum Pezizomycotina [10,34]. *ace1*, repressor of cellulase and xylanase genes in *Hypocrea jecorina* (*T*. *reesei*) [35], was the only characterized homolog of SltA, with high conservation of the DNA-binding domain and both SltA and ACE1 recognizing the consensus sequence 5′-AGGCA-3′ [10,35].

*C*. *gloeosporioides* SltA encodes a 775-residue protein containing, as in *Aspergillus* and *Trichoderma*, three C_2_H_2_ zinc fingers that constitute the DNA-binding domain of this transcription factor. *C*. *gloeosporioides* SltA analysis showed that the highest conserved regions are the zinc-finger domain and the adjacent amino acids, including tryptophan 502 (W502), whose codon was changed to a stop codon in the *A*. *nidulans sltA1* mutant [10]. However, the presence of additional conserved motifs in the C- and N-terminal regions may well indicate conservation of possible regulatory domains and functions for all members of this family of transcription factors. Recently, a second element of the Slt pathway was described in *A*. *nidulans*—SltB—the deletion of which caused a defect in cation-stress tolerance similar to that displayed by the null *sltA* strain [6]. Recently, SltB-mediated proteolytic activation of SltA has been described [36]. A truncated 32-kD version of SltA, comprising the DNA-binding and conserved C-terminal motifs, is sufficient to mediate cation homeostasis in *A*. *nidulans*. The presence of a SltB homolog in *C*. *gloeosporioides* supports a similar mode of SltA post-translational modification in this fungus.

*C*. *gloeosporioides* CrzA encodes a 682-amino acid residue protein containing three conserved zinc-finger domains. The *S*. *cerevisiae* Crz1p contains a zinc-finger DNA-binding motif that specifically binds to a 24-bp sequence in gene promoter regions, the calcineurin-dependent response element [13]. Multiple sequence analysis of CrzA proteins of *C*. *gloeosporioides*, *A*. *nidulans A*. *fumigatu*s, *N*. *crassa*, *Chaetomium globosum*, *Coccidioides immitis*, *Phaeosphaeria nodorum* and *S*. *cerevisiae* showed the presence of three conserved C_2_H_2_ transcription factor domains. It also revealed the presence two putative conserved CDDs required in calcineurin-dependent regulation. In yeast, Crz1p CDDs help in nuclear export and activity [37]. Furthermore, altering the PxIxIT motif of CDDs to PVIVIT in Crz1 of yeast resulted in growth defects during alkaline stress by manipulating calcineurin away from other substrates or regulators [38]. In *A*. *nidulans*, deletion of CDD2 precludes the CrzA—calcineurin interaction and corrects intracellular trafficking of this transcription factor in the presence of calcium or alkaline pH stress [30]. The sequence similarities in the concerned zinc-finger domain and CDD indicate their possible similarity in roles for CrzA in *C*. *gloeosporioides*.

### The phenotype of the *crzA* and *sltA* knockouts

Knockout of *crzA* has a wide morphogenetic effect on growth, sporulation, germination and inhibition of appressorium formation and pathogenicity of *Colletotrichum*. The Δ*crzA* mutant of *A*. *fumigatus* shows defective growth and germination, as well as diminished virulence in mice [14,39,40], similar to the *C*. *gloeosporioides crzA* in the present study on fruit. In *Aspergillus parasiticus*, CrzA is required for growth and aflatoxin biosynthesis under conditions of calcium stress [30,41], since it modulates cell toxicity, something that is not observed in *Colletotrichum*. The strong morphogenetic effects are probably the result of inhibition of chitin biosynthesis, likely due to inhibition of class III and V chitin synthases [10] as confirmed by low CFW staining in the null crzA mutant, shown in S7 Fig. Similar responses have been observed in null mutants of *S*. *cerevisiae* [13] and *A*. *nidulans*, where reduced expression of cell wall-synthesis genes such as *chsA*, *chsB* and *fsk1A* was found in *C*. *gloeosporioides* (Fig 12) and is required for the maintenance of cell-wall integrity [10,16]. However, the present results differ from those of Dinamarco *et al*. [42], which suggested that the *crzA* mutant modulates Ca^2+^ transporters, given that Ca^2+^ treatment of *Colletotrichum* enhanced fungal growth density but only slightly reduced the morphogenetic development and did not improve pathogenicity (Figs 5 and 11). This suggests that CrzA may have wider functions than those reported for this transcription factor in *Aspergillus*.

In addition, the behavior of transcription factor SltA in *Colletotrichum* was completely different from that in *A*. *nidulans*. Whereas in *Aspergillus*, *sltA* was reported to affect cation adaptation and homoeostasis during fungal growth, in *Colletotrichum*, appressorium formation from germinated spores was significantly affected. The inhibition of appressorium formation occurred concurrently with downregulation of the MAPK *pmk1* and *cat1*, shown to have roles in appressorium-formation processes as described for *Magnaporthe* [27]. This MAPK modulates the mobilization of lipids and glycogen [43], accompanied by an increase in triacylglycerol activity, which liberates glycerol from stored lipids in germinated spores [27]. In addition, *cat1* contributes to fatty acid beta-oxidation and abnormal chitin distribution which have a potential role in appressorium formation and penetration [44,45].

Interestingly, growth colonization of *crzA* and *sltA* were accompanied by minor color changes of growth in cultures. While few reports, if any, have indicated the secretion of specific complex secondary metabolites by this *Colletotrichum* in culture [46], some simple metabolites have been found to be secreted by this fungus including ammonia and gluconic acid [1]. However, none of those compounds were found to be induced under stress conditions by salts.

### Cation homeostasis modulated by *crzA* and *sltA* and restoration by addition of Ca^2+^

Cation homeostasis is regulated by a complex network of transporters and their regulators [47], including the proton-pumping ATPase Pma1 [48], the K^+^ transporters Trk1 and Trk2 [49] which pump in large amounts of K^+^, and the Na^+^ exporter Ena1 [50,51]. SltA and CrzA are also known for their positive role in preventing the toxicity of cations such as Na^+^, K^+^, Li^+^, Cs^+^ and Mg^2+^, but not Ca^2+^ [10,11,32,52]. Spielvogel *et al*. [10] suggested that *sltA* can bind DNA, positively modulating transcription reduction of the *ena1*-like sodium pump (*enaA*, a putative cation exporter), and negatively affecting transcription of the putative vacuolar Ca^2+^/H^+^ exchanger *vcxA*, enabling a detoxification response [53,54]. In addition, the CrzA transcription factor activates the expression of various target genes [55], including those that encode cation transporters that act at the plasma membrane or in other membrane organelles [13,56,57].

In *C*. *gloeosporioides*, both transcription factors modulated the differential toxicity of K^+^, Na^+^, and Li^+^, but they were not affected by Ca^2+^, as reported for *Aspergillus*. The *sltA* mutants displayed normal growth in the presence of 25 mM KCl or NaCl and 5 mM LiCl, but 30–90% inhibition of sporulation, germination, and appressorium formation. However, the drastic morphogenetic effect of the *crzA* deletion on growth, sporulation, germination and appressorium formation of *C*. *gloeosporioides*, which reached up to 95%, did not leave much space for a further effect of cation toxicity.

Ca^2+^ restoration of morphogenetic effects was mainly observed in the induction of appressorium formation of *sltA* mutants, where the 40–50% inhibition of appressorium formation was almost fully restored. This occurred in parallel to restoration of the relative expression of *pmk1* and *cat1*, suggesting their dependence on Ca^2+^ regulation. Transcript levels of the putative vacuolar Ca^2+^/H^+^ exchanger gene *vcxA* in *Aspergillus* in response to high Ca^2+^ levels provide a possible explanation for the lack of Ca^2+^ toxicity in Δ*sltA* strains. These strains show elevation of *vcxA* transcript levels in response to Ca^2+^, putatively allowing greater vacuolar storage and thus detoxification of Ca^2+^. Excess Ca^2+^ may activate appressorium formation (Fig 7) directly via a Ca^2+^/CaM-inducing system, as found in *Zoophthora radicans* [58], or indirectly by activation of MAPK signaling pathways, as described in *Magnaporthe* and presently in *C*. *gloeosporioides* [27].

Ca^2+^ treatments, however, showed only minor restoration from cation toxicity in the *crzA* mutants. This may be because CrzA is the final effector of the calcium-signaling pathway in this fungus. This suggests that while Ca^2+^ could repair processes in the Δ*sltA* background, it could not do so in Δ*crzA*, suggesting broad regulation of this transcription factor in *C*. *gloeosporioides*. Absence of CrzA function may limit or completely inhibit the expression of genes whose products are required for calcium homeostasis and calcium-dependent response, such as those studied here for adequate cell-wall construction or fungal virulence.

### *crzA* and *sltA* affects pathogenicity

Genes activated by Crz1/CrzA include calcium channels and transporters that import calcium into vacuoles or export it [42]. Aside from calcium transporters, the hexose transporter *HXT3* [59], the glycosylphosphatidylinositol-linked aspartyl protease *Yps1* [60], *chsB*, the *Aspergillus giganteus* antifungal protein-encoding *afp* [10], the calcium-binding protein *CBP1* [61], and recently CrzA, have been shown to directly control transcription of the calcium transporter genes *pmcA—C* [42]. All of this might explain the extreme sensitivity of the morphogenetic stages, such as sporulation, germination, appressorium formation and pathogenicity induced by the *crzA* deletion, resulting in markedly aberrant morphogenetic effects. Inhibition of appressorium formation in the *ΔsltA* mutants of *C*. *gloeosporioides* also explains the reduced colonization of *Colletotrichum*, *Magnaporthe* and others that breach the host cuticle via appressorium formation.

These findings suggest new targets for fungicide development, i.e., compounds that modulate calcium channels and transporters involved in the import or efflux of calcium into vacuoles. Treatments of mango fruit discs in the presence of 1.5 mM KCl or NaCl both inhibited colonization and symptom development of anthracnose caused by *C*. *gloeosporioides*. These findings suggest that the differential sensitivity to cations might be used for disease control in fruit by simple cation treatments. Furthermore, expression of the calcineurin catalytic subunit is independent of CrzA transcriptional activity in *C*. *gloeosporioides*. However, it is a direct target for antifungals because it is a well-known virulence factor [32] and, in this fungus, specific inhibition of calcineurin activity may decrease or completely inhibit CrzA activity and may have consequent effects on pathogenicity [32, 62]. Present fungicide targets include sterol biosynthesis, respiration, multistage biosynthetic processes and others [15]. However, alteration of the cation transporter has rarely been suggested as a possible modulator of fungal growth and appressorium formation. The possibility of modulating specific ion transporters that affect cation toxicity and inhibit MAPK signaling may be an interesting target for future development of specific fungal inhibitors.

## Conclusions

The relative sensitivity of fungal pathogens to cations was considered in this work for a first possible approach to fungal inhibition of pathogens in postharvest fruit. Homologs of *A*. *nidulans* SltA and CrzA that modulate cation homeostasis in fungi were identified in *C*. *gloeosporioides* and found to modulate morphogenetic pathways (sporulation, germination, appressorium formation and colonization) in the latter. Whereas *ΔcrzA* showed a drastic morphogenetic response that could only be partially restored by Ca^2+^, *slt*A knockout mainly reduced appressorium formation; this was correlated with downregulation of *pmk1* and *cat1*, which modulate appressorium formation. The functionality of Ca^2+^ in restoring *pmk1* and *cat1* expression, appressorium formation and pathogen colonization on avocado and mango fruit suggests Ca^2+^-dependence of *sltA* in *C*. *gloeosporioides*. However, whereas the *ΔcrzA* mutant showed a drastic response in terms of fungal growth, sporulation, germination and appressorium formation, these were only slightly restored by Ca^2+^ treatment. As in *A*. *nidulans*, elevated concentrations of Na^+^, K^+^, and Li^+^ showed higher toxicity to the *ΔsltA* and *ΔcrzA* mutants. However, both mutants had significantly inhibited fruit colonization. It would be interesting to investigate how both pathways act in concert. Parallel or convergent pathways are possible, as are similar targets or shared cofactors or coregulators.

## Supporting Information

S1 Fig Multiple sequence alignment of zinc-finger transcription factor SltA from *Aspergillus nidulans* (AN2919, SltA), *Trichoderma reesei* (Tr_ACE1), *Colletotrichum gloeosporioides*-5 (Cg5_SltA) and *Colletotrichum gloeosporioides* strain CG-14 (Cg14_SltA) showing the gene code, putative translation start site for SltA and comparison with *A*. *nidulans* SltA and ACE1. Fully conserved residues are highlighted in dark blue, 60% conserved residues in blue, 30% conserved residues in light blue and non-conserved residues have a white background. Red boxes indicate the limits for boundaries of the three zinc fingers (zf). In yellow are Cys and His residues putatively involved in chelating zinc atoms.
(TIF)

S2 Fig Multiple alignment of the zinc-finger domains of predicted CrzA homologs from different filamentous fungi. The zinc-finger region is shown (red boxes), with classical C_2_H_2_ zinc fingers zf1 and zf2, and non-canonical CCHC zinc finger zf3. Protein accession numbers are as follows: Cglo-14, *C*. *gloesporoides* EQB55543.1; Anid, *A*. *nidulans* BAE94327; Afum, *A*. *fumigatu*s EAL88401; Ncra, *N*. *crassa* EAA32849; Mory, XP_359644.1 *Magnaporthe oryzae*; Cglb, *Chaetomium globosum* EAQ88414; Cimm, *Coccidioides immitis* EAS33001; Pnod, *Parastagonospora nodorum* EAT87393. Alignment of a select number of Crz1/CrzA homologs shows the putative calcineurin-docking domains (CDDs, green boxes). Protein alignments were performed using Clustal Omega online service at EBI (http://www.ebi.ac.uk/Tools/msa/clustalo/). Residue shading as in legend of S1 Fig.
(TIF)

S3 Fig Double-crossover homologous recombination event resulting in replacement of the original *sltA*/*crzA* sequence with the *sltA/crzA-5'-Hyg-sltA/crzA-3'* cassette. (A) Scheme describing gene disruption by homologous recombination. The pairs of primers used to create the construct were attBsltA/*crzA* _5'F/–attBsltA/*crzA* _5'R for the 5' end and attBsltA/*crzA* _3'F—attBsltA/*crzA* _3'R for the 3' end. (B) PCR analysis of the WT strain, ectopic colony (Ect), and independent *sltA*-disrupted colonies *(ΔsltA*). (C) PCR analysis of the WT strain, ectopic colony (Ect), and independent *crzA*-disrupted colonies *(ΔcrzA*). sltA/crzA _5’ctrl_F (S1 Table) flanking a position upstream of the *sltA*:*HYG3* region and reverse primer Hyg_5'R (S1 Table) located on the hygromycin cassette were used to identify positive *sltA/crzA* gene replacement at the 5′ locus. Hyg_3'F (S1 Table) from the hygromycin cassette and sltA/crzA _3’ctrl_R (S1 Table) flanking the *sltA*:*HYG3* region were used to identify sltA/crzA gene replacement at the 3′ locus. sltA/crzA attB primers for the 5' and 3' ends (S1 Table) were used for WT DNA quality control (not shown). Primers attB sltA/crzA _5'F and Hyg_5'R (S1 Table) were used as a positive control for the ectopic strains, to confirm random integration of the *5'- sltA/crzA*:*HYG3* cassette. (D) Relative expression of *ΔsltA*, WT and ectopic-integration strains, as detected by qRT-PCR. The relative expression values obtained by qRT-PCR were normalized against *18S* rRNA. Values represent means ± SE of duplicates. (E) Relative expression of WT strain, *ΔcrzA* and ectopic-integration strains, as detected by qRT-PCR. The relative expression values obtained by qRT-PCR were normalized against *18S* rRNA. Experiments were repeated three times and results of a single representative experiment are shown. Average of three technical replications is presented and asterisks marked columns are significantly different at P ≤ 0.05 according to the Student’s t-test.
(TIF)

S4 Fig Growth, sporulation, germination and appressorium formation by the wild-type (WT), ectopic (Ect) and mutant strains of *Δcrz*A (*ΔcrzA31A*, *ΔcrzA36A* and Ect*crzA37B*) and *Δslt*A (*ΔsltA10A*, *ΔsltA21C* and Ect*sltA1C*) in glucose minimal media amended (□), or not (■), with NaCl. These *C*. *gloeosporioides* strains were disc-inoculated on glucose minimal media amended with 25 mM NaCl and incubated for 5 days at 24°C. (A, E) Radial colony growth, (B, F) sporulation, (C, G) germination and (D, H) appressorium formation were evaluated after 16 h of incubation on glass slides at 24°C. Experiments were repeated three times and results of a single representative experiment are shown. Columns with different letters (lower or upper case) are significantly different at P ≤ 0.05 according to the Tukey-Kramer multiple comparison test.
(TIF)

S5 Fig Growth, sporulation, germination and appressorium formation by the wild-type (WT), ectopic (Ect) and mutant strains of *Δcrz*A (*ΔcrzA31A*, *ΔcrzA36A* and Ect*crzA37B*) and *Δslt*A (*ΔsltA10A*, *ΔsltA21C* and Ect*sltA1C*) in glucose minimal media amended (□), or not (■), with LiCl. These *C*. *gloeosporioides* strains were disc-inoculated on glucose minimal media amended with 25 mM LiCl and incubated for 5 days at 24°C. (A, E) Radial colony growth, (B, F) sporulation, (C, G) germination and (D, H) appressorium formation were evaluated after 16 h of incubation on glass slides at 24°C. Experiments were repeated three times and one of the experiments is reported. Experiments were repeated three times and results of a single representative experiment are shown. Columns with different letters (lower or upper case) are significantly different at P ≤ 0.05 according to the Tukey-Kramer multiple comparison test.
(TIF)

S6 Fig Appressorium formation by spores of *C*. *gloeosporioides* WT, *ΔsltA21C*, *ΔsltA10A* and Ect*sltA1C* strains in the presence of water (□) or 10 mM CaCl_2_ (■). Five drops of spores of the different strains were placed on the peel of mango fruit cv. Shely and incubated in a humid plastic container overnight at 24°C. Microscopic evaluation of germination and appressorium formation was evaluated after the 0.3–0.5 mm thick peel containing the inoculated drops was excised from the fruit 24 h after inoculation. Experiments were repeated three times and results of a single representative experiment are shown. Columns with different letters (lower or upper case) are significantly different at P ≤ 0.05 according to the Tukey-Kramer multiple comparison test.
(TIF)

S7 Fig Calcofluor white staining of the mycelia of the WT (a) and *Δ*crzA31a (b) strain as observed by Fluorescent Micrsocopy. Microscopic evaluation indicate the reduced staining of the ΔcrzA31a mutant because of the downregulation of chitin synthesis.
(TIF)

S1 Table Primers used in this research. (DOCX)

## References

[1] Bi F, Barad S, Ment D, Luria N, Dubey A, Casado V, et al Carbon regulation of environmental pH by secreted small molecules that modulate pathogenicity in phytopathogenic fungi. Mol Plant Pathol. 2016; 17: 1178–1195. 26666972 10.1111/mpp.12355 PMC6638356

[2] Ment D, Alkan N, Luria N, Bi FC, Reuveni E, Fluhr R, et al A Role of AREB in the Regulation of PACC-Dependent Acid-Expressed-Genes and Pathogenicity of Colletotrichum gloeosporioides. Mol Plant Microbe Interact. 2015; 28: 154–166. 10.1094/MPMI-09-14-0252-R 25317668

[3] Barad S, Horowitz SB, Kobiler I, Sherman A, Prusky D. Accumulation of the mycotoxin patulin in the presence of gluconic acid contributes to pathogenicity of Penicillium expansum. Mol Plant Microbe Interact. 2014; 27: 66–77. 10.1094/MPMI-05-13-0138-R 24024763

[4] Miyara I, Shafran H, Kramer Haimovich H, Rollins J, Sherman A, et al Multi-factor regulation of pectate lyase secretion by Colletotrichum gloeosporioides pathogenic on avocado fruits. Mol Plant Pathol. 2008; 9: 281–291. 18705870 10.1111/j.1364-3703.2007.00462.x PMC6640356

[5] Shantappa S, Dhingra S, Hernandez-Ortiz P, Espeso EA, Calvo AM. Role of the zinc finger transcription factor SltA in morphogenesis and sterigmatocystin biosynthesis in the fungus Aspergillus nidulans. PLoS One. 2013; 8: e68492 10.1371/journal.pone.0068492 23840895 PMC3698166

[6] Mellado L, Calcagno-Pizarelli AM, Lockington RA, Cortese MS, Kelly JM, Arst HN Jr, et al A second component of the SltA-dependent cation tolerance pathway in Aspergillus nidulans. Fungal Genet Biol. 2015; 82: 116–128. 10.1016/j.fgb.2015.06.002 26119498 PMC4557415

[7] Tournas V. Heat-resistant fungi of importance to the food and beverage industry. Crit Rev Microbiol. 1994; 20: 243–263. 10.3109/10408419409113558 7857517

[8] Asrar A-WA, Elhindi KM. Alleviation of drought stress of marigold (Tagetes erecta) plants by using arbuscular mycorrhizal fungi. Saudi J Biol Sci. 2011; 18: 93–98. 10.1016/j.sjbs.2010.06.007 23961109 PMC3730742

[9] Prusky D, Alkan N, Mengiste T, Fluhr R. Quiescent and necrotrophic lifestyle choice during postharvest disease development. Annu Rev Phytopathol. 2013; 51: 155–176 10.1146/annurev-phyto-082712-102349 23682917

[10] Spielvogel A, Findon H, Arst HN, Araujo-Bazan L, Hernandez-Ortiz P, Stahl U, et al Two zinc finger transcription factors, CrzA and SltA, are involved in cation homoeostasis and detoxification in Aspergillus nidulans. Biochem J. 2008; 414: 419–429. 10.1042/BJ20080344 18471095

[11] Spathas DH. A salt sensitive mutation on chromosome VI of Aspergillus nidulans. Aspergillus Newsletter. 1978; 46: 28.

[12] Calcagno-Pizarelli AM, Hervás-Aguilar A, Galindo A, Abenza JF, Peñalva MA, Arst HN, et al Rescue of Aspergillus nidulans severely debilitating null mutations in ESCRT-0, I, II and III genes by inactivation of a salt-tolerance pathway allows examination of ESCRT gene roles in pH signalling. J Cell Sci. 2011; 124: 4064–4076. 10.1242/jcs.088344 22135362 PMC3244986

[13] Stathopoulos AM, Cyert MS. Calcineurin acts through the CRZ1/TCN1-encoded transcription factor to regulate gene expression in yeast. Genes Dev. 1997; 11(24): 3432–3444. 9407035 10.1101/gad.11.24.3432 PMC316814

[14] Soriani FM, Malavazi I, da Silva Ferreira ME, Savoldi M, Von Zeska Kress MR, de Souza Goldman MH, et al Functional characterization of the Aspergillus fumigatus CRZ1 homologue, CrzA. Mol Microbiol. 2008; 67: 1274–1291. 18298443 10.1111/j.1365-2958.2008.06122.x

[15] Juvvadi PR, Lamoth F, Steinbach WJ. Calcineurin as a multifunctional regulator: unraveling novel functions in fungal stress responses, hyphal growth, drug resistance, and pathogenesis. Fungal Biol Rev. 2014; 28: 56–69. 10.1016/j.fbr.2014.02.004 25383089 PMC4219591

[16] Hagiwara D, Kondo A, Fujioka T, Abe K. Functional analysis of C2H2 zinc finger transcription factor CrzA involved in calcium signaling in Aspergillus nidulans. Curr genet. 2008; 54: 325–338. 10.1007/s00294-008-0220-z 19002465

[17] Garzia A, Etxebeste O, Herrero-García E, Ugalde U, Espeso EA. The concerted action of bZip and cMyb transcription factors FlbB and FlbD induces brlA expression and asexual development in Aspergillus nidulans. Mol Microbiol. 2010; 75: 1314–1324. 20132447 10.1111/j.1365-2958.2010.07063.x

[18] Prusky D, Alkan N, Miyara I, Barad S, Davidzon M, Kobiler I, et al Mechanisms modulating postharvest pathogen colonization of decaying fruits In: Prusky D, Gullino ML, editors. Postharvest Pathology Plant pathology in the 21st Century, Contributions to the 10th International Congress, ICPP 2013 volume 7 Springer: 2014 pp. 43–55.

[19] Tu JC. An improved mathur's medium for growth sporulation and germination of spores of Colletotrichum lindemuthianum. Microbios. 1985; 44: 87–93.

[20] Miyara I, Shafran H, Davidzon M, Sherman A, Prusky D. pH Regulation of ammonia secretion by Colletotrichum gloeosporioides and its effect on appressorium formation and pathogenicity. Mol Plant Microbe Interact. 2010; 23: 304–316. 10.1094/MPMI-23-3-0304 20121452

[21] Käfer E. Meiotic and mitotic recombination in Aspergillus and its chromosomal aberrations. Adv Genet. 1977; 19: 33–131. 327767 10.1016/s0065-2660(08)60245-x

[22] Miyara I, Shafran H, Davidzon M, Sherman A, Prusky D. pH regulation of ammonia secretion by Colletotrichum gloeosporioides and its effect on appressorium formation and pathogenicity. Mol Plant Microbe Interact. 2010; 23: 304–316. 10.1094/MPMI-23-3-0304 20121452

[23] Shafran H, Miyara I, Eshed R, Prusky D, Sherman A. Development of new tools for studying gene function in fungi based on the Gateway system. Fungal Genetics and Biology. 2008; 45: 1147–1154. 10.1016/j.fgb.2008.04.011 18550398

[24] Yakoby N, Beno-Moualem D, Keen NT, Dinoor A, Pines O, Prusky D. Colletotrichum gloeosporioides pelB is an important virulence factor in avocado fruit-fungus interaction. Mol Plant Microbe Interact. 2001; 14: 988–995. 10.1094/MPMI.2001.14.8.988 11497471

[25] Robinson M, Sharon A. Transformation of the bioherbicide Colletotrichum gloeosporioides f. sp. Aeschynomene by electroporation of germinated conidia. Curr Genet. 1999; 36: 98–104. 10447601 10.1007/s002940050478

[26] Barhoom S, Sharon A. cAMP regulation of "pathogenic" and "saprophytic" fungal spore germination. Fungal Genet Biol. 2004; 41: 317–326. 10.1016/j.fgb.2003.11.011 14761792

[27] Soanes DM, Chakrabarti A, Paszkiewicz KH, Dawe AL, Talbot NJ. Genome-wide transcriptional profiling of appressorium development by the rice blast fungus Magnaporthe oryzae. Plos Pathogens. 2012; 2; 8(2):e1002514 10.1371/journal.ppat.1002514 22346750 PMC3276559

[28] Livak KJ, Schmittgen TD. Analysis of relative gene expression data using real-time quantitative PCR and the 2−ΔΔCT method. Methods. 2001; 25 (4): 402–408. 10.1006/meth.2001.1262 11846609

[29] Harrington BJ, Hageage GJ. Calcofluor White: A review of its uses and applications in clinical mycology and parasitology. Laboratory Medicine. 2003; 34: 361–367.

[30] Hernández-Ortiz P, Espeso EA. Phospho-regulation and nucleocytoplasmic trafficking of CrzA in response to calcium and alkaline-pH stress in Aspergillus nidulans. Mol Microbiol. 2013; 89: 532–551. 23772954 10.1111/mmi.12294

[31] Abang MM, Abraham W-R, Asiedu R, Hoffmann P, Wolf G, Winter S. Secondary metabolite profile and phytotoxic activity of genetically distinct forms of Colletotrichum gloeosporioides from yam (Dioscorea spp.). Mycol Res. (2009); 113: 130–140. 10.1016/j.mycres.2008.09.004 18929651

[32] Findon H, Calcagno-Pizarelli AM, Martinez JL, Spielvogel A, Markina-Inarrairaegui A, Indrakumar T, et al Analysis of a novel calcium auxotrophy in Aspergillus nidulans. Fungal Genet Biol. 2010; 47: 647–655. 10.1016/j.fgb.2010.04.002 20438880 PMC2884188

[33] Stathopoulos-Gerontides A, Guo JJ, Cyert MS. Yeast calcineurin regulates nuclear localization of the Crz1p transcription factor through dephosphorylation. Genes Dev. 1999; 13: 798–803. 10197980 10.1101/gad.13.7.798 PMC316598

[34] Chilton IJ, Delaney CE, Barham-Morris J, Fincham DA, Hooley P, Whitehead MP, et al The Aspergillus nidulans stress response transcription factor StzA is ascomycete-specific and shows species-specific polymorphisms in the C-terminal region. Mycol Res. 2008; 112: 1435–1446. 10.1016/j.mycres.2008.06.028 18678248

[35] Saloheimo A, Aro N, Ilmen M, Penttila M. Isolation of the ace1 gene encoding a Cys(2)-His(2) transcription factor involved in regulation of activity of the cellulase promoter cbh1 of Trichoderma reesei. J Biol Chem. 2000; 275: 5817–5825. 10681571 10.1074/jbc.275.8.5817

[36] Mellado L, Arst HN, Espeso EA. Proteolytic activation of both components of the cation stress—responsive Slt pathway in Aspergillus nidulans. Mol Biol Cell. 2016; 27: 2598–2612. 10.1091/mbc.E16-01-0049 27307585 PMC4985261

[37] Boustany LM, Cyert MS. Calcineurin-dependent regulation of Crz1p nuclear export requires Msn5p and a conserved calcineurin docking site. Genes Dev. 2002; 16: 608–619. 10.1101/gad.967602 11877380 PMC155349

[38] Roy J, Li H, Hogan PG, Cyert MS. A conserved docking site modulates substrate affinity for calcineurin, signaling output, and in vivo function. Mol Cell. 2007; 25: 889–901. 10.1016/j.molcel.2007.02.014 17386265 PMC2913616

[39] Cramer RA, Perfect BZ, Pinchai N, Park S, Perlin DS, Asfaw YG, et al Calcineurin target CrzA regulates conidial germination, hyphal growth, and pathogenesis of Aspergillus fumigatus. Eukaryot Cell. 2008; 7 (7): 1085–1097. 10.1128/EC.00086-08 18456861 PMC2446674

[40] Perrin RM, Fedorova ND, Bok JW, Cramer RA Jr, Wortman JR, Kim HS, et al Transcriptional regulation of chemical diversity in Aspergillus fumigatus by LaeA. PLoS Pathog. 2007 3 (4): e50 10.1371/journal.ppat.0030050 17432932 PMC1851976

[41] Chang PK. Aspergillus parasiticus crzA, which encodes calcineurin response zinc-finger protein, is required for aflatoxin production under calcium stress. Int J Mol Sci. 2008; 9: 2027–2043. 10.3390/ijms9102027 19325734 PMC2635607

[42] Dinamarco TM, Freitas FZ, Almeida RS, Brown NA, dos Reis TF, Ramalho LN, et al Functional characterization of an Aspergillus fumigatus calcium transporter (PmcA) that Is essential for fungal infection. PLoS One. 2012; 7 (5): e37591 10.1371/journal.pone.0037591 22649543 PMC3359301

[43] Thines E, Weber RWS, Talbot NJ. MAP kinase and protein kinase A-dependent mobilization of triacylglycerol and glycogen during appressorium turgor generation by Magnaporthe grisea. Plant Cell. 2000; 12 (9): 1703–1718. 11006342 10.1105/tpc.12.9.1703 PMC149080

[44] Bhambra GK, Wang ZY, Soanes DM, Wakley GE, Talbot NJ. Peroxisomal carnitine acetyl transferase is required for elaboration of penetration hyphae during plant infection by Magnaporthe grisea. Mol Microbio. 2006; 61 (1): 46–60. 10.1111/j.1365-2958.2006.05209.x 16824094

[45] Ramos-Pamplona M, Naqvi NI. Host invasion during rice-blast disease requires carnitine-dependent transport of peroxisomal acetyl-CoA. Mol Microbiol. 2006; 61 (1): 61–75. 16824095 10.1111/j.1365-2958.2006.05194.x

[46] Pusztahelyi T, Holb IJ, Pócsi I. Secondary metabolites in fungus-plant interactions. Frontiers in Plant Science. 2015; 6: 573 10.3389/fpls.2015.00573 26300892 PMC4527079

[47] Rodríguez-Navarro A, Rubio F. High-affinity potassium and sodium transport systems in plants. J Exp Bot. 2006; 57: 1149–1160. 10.1093/jxb/erj068 16449373

[48] Ferreira T, Mason AB, Slayman CW. The yeast Pma1 proton pump: a model for understanding the biogenesis of plasma membrane proteins. Journal of Biological Chemistry. 2001; 276: 29613–29616. 10.1074/jbc.R100022200 11404364

[49] Gaber RF. Molecular genetics of yeast ion transport In: Martin F, Michael M, editors. Int Rev Cytol. Academic Press 1992, 12 31;137:; pp. 299–353. 1330965 10.1016/s0074-7696(08)62679-0

[50] Haro R, Bañuelos MA, Quintero FJ, Rubio F, Rodríguez-Navarro A. Genetic basis of sodium exclusion and sodium tolerance in yeast. A model for plants. Physiol Plant. 1993; 89: 868–874.

[51] Wieland J, Nitsche AM, Strayle J, Steiner H, Rudolph HK. The PMR2 gene cluster encodes functionally distinct isoforms of a putative Na+ pump in the yeast plasma membrane. Embo J. 1995; 14: 3870–3882. 7664728 10.1002/j.1460-2075.1995.tb00059.x PMC394466

[52] O'Neil JD, Bugno M, Stanley MS, Barham-Morris JB, Woodcock NA, Clement DJ, et al Cloning of a novel gene encoding a C2H2 zinc finger protein that alleviates sensitivity to abiotic stresses in Aspergillus nidulans. Mycol Res. 2002; 106: 491–498.

[53] Chen S, Song Y, Cao J, Wang G, Wei H, Xu X, et al Localization and function of calmodulin in live-cells of Aspergillus nidulans. Fungal Genet Biol. 2010; 47 (3): 268–278. 10.1016/j.fgb.2009.12.008 20034586

[54] Pitt D, Barnes J. Calcium homeostasis, signalling and protein phosphorylation during calcium-induced conidiation in Penicillium notatum. J Gen Microbiol. 1993; 139: 3053–3063. 10.1099/00221287-139-12-3053 8126432

[55] Yoshimoto H, Saltsman K, Gasch AP, Li HX, Ogawa N, Botstein D, et al Genome-wide analysis of gene expression regulated by the calcineurin/Crz1p signaling pathway in Saccharomyces cerevisiae. J Biol Chem. 2002; 277: 31079–31088. 10.1074/jbc.M202718200 12058033

[56] Serrano M, Bayon J, Pascolo L, Tiribelli C, Ostrow J, Gonzalez-Gallego J, et al Evidence for carrier-mediated transport of unconjugated bilirubin across plasma membrane vesicles from human placental trophoblast. Placenta. 2002; 23: 527–535. 12175967 10.1053/plac.2002.0838

[57] Matheos DP, Kingsbury TJ, Ahsan US, Cunningham KW. Tcn1p/Crz1p, a calcineurin-dependent transcription factor that differentially regulates gene expression in Saccharomyces cerevisiae. Genes Dev. 1997; 11: 3445–3458. 9407036 10.1101/gad.11.24.3445 PMC316804

[58] Magalhaes B, Wayne R, Humber R, Shields E, Roberts D. Calcium-regulated appressorium formation of the entomopathogenic fungus Zoophthora radicans. Protoplasma. 1991; 160: 77–88.

[59] Ruiz A, Serrano R, Arino J. Direct regulation of genes involved in glucose utilization by the calcium/calcineurin pathway. J Biol Chem. 2008; 283: 13923–13933. 10.1074/jbc.M708683200 18362157

[60] Miyazaki T, Izumikawa K, Yamauchi S, Inamine T, Nagayoshi Y, Saijo T, et al The glycosylphosphatidylinositol-linked aspartyl protease Yps1 is transcriptionally regulated by the calcineurin-Crz1 and Slt2 MAPK pathways in Candida glabrata. FEMS Yeast Res. 2011; 11: 449–456. 10.1111/j.1567-1364.2011.00734.x 21501380

[61] Kim S, Hu J, Oh Y, Park J, Choi J, Lee YH, et al Combining ChIP-chip and expression profiling to model the MoCRZ1 mediated circuit for Ca/calcineurin signaling in the rice blast fungus. PLoS Pathog. 2010; 6: e1000909 10.1371/journal.ppat.1000909 20502632 PMC2873923

[62] Thewes S. Calcineurin-Crz1 signaling in lower eukaryotes. Eukaryot cell. 2014;13 (6): 694–705. 10.1128/EC.00038-14 24681686 PMC4054267

