# Supplementary figures and images for: Cation-Stress-Responsive Transcription Factors SltA and CrzA Regulate Morphogenetic Processes and Pathogenicity of Colletotrichum gloeosporioides
Source: PLoS One. 2016 Dec 28;11(12):e0168561. doi: 10.1371/journal.pone.0168561 (PMC5193415; doi:10.1371/journal.pone.0168561)

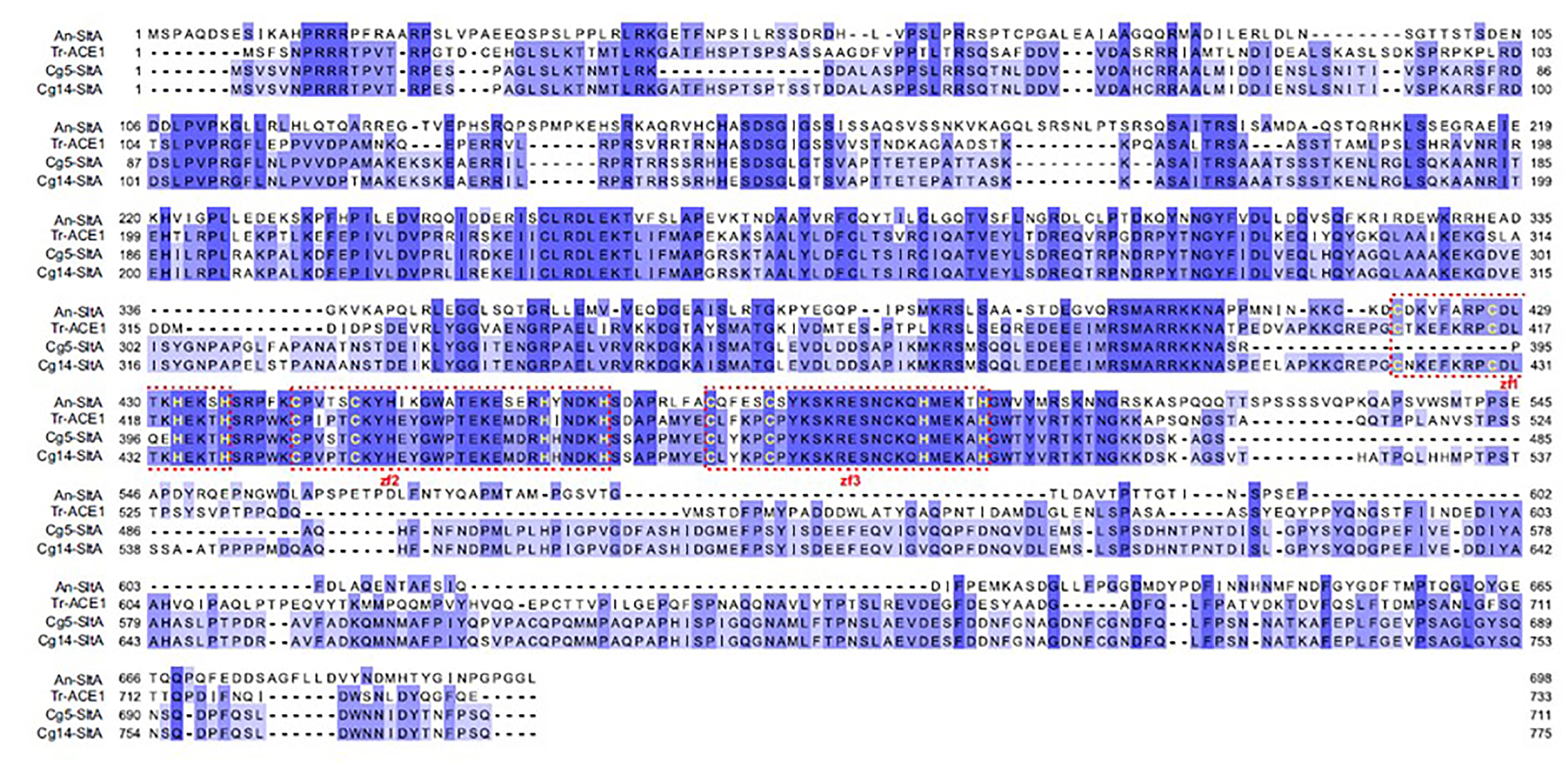

Supplement: S1 Fig — Fully conserved residues are highlighted in dark blue, 60% conserved residues in blue, 30% conserved residues in light blue and non-conserved residues have a white background. Red boxes indicate the limits for boundaries of the three zinc fingers (zf). In yellow are Cys and His residues putatively involved in chelating zinc atoms. (TIF) [file pone.0168561.s001.tif]

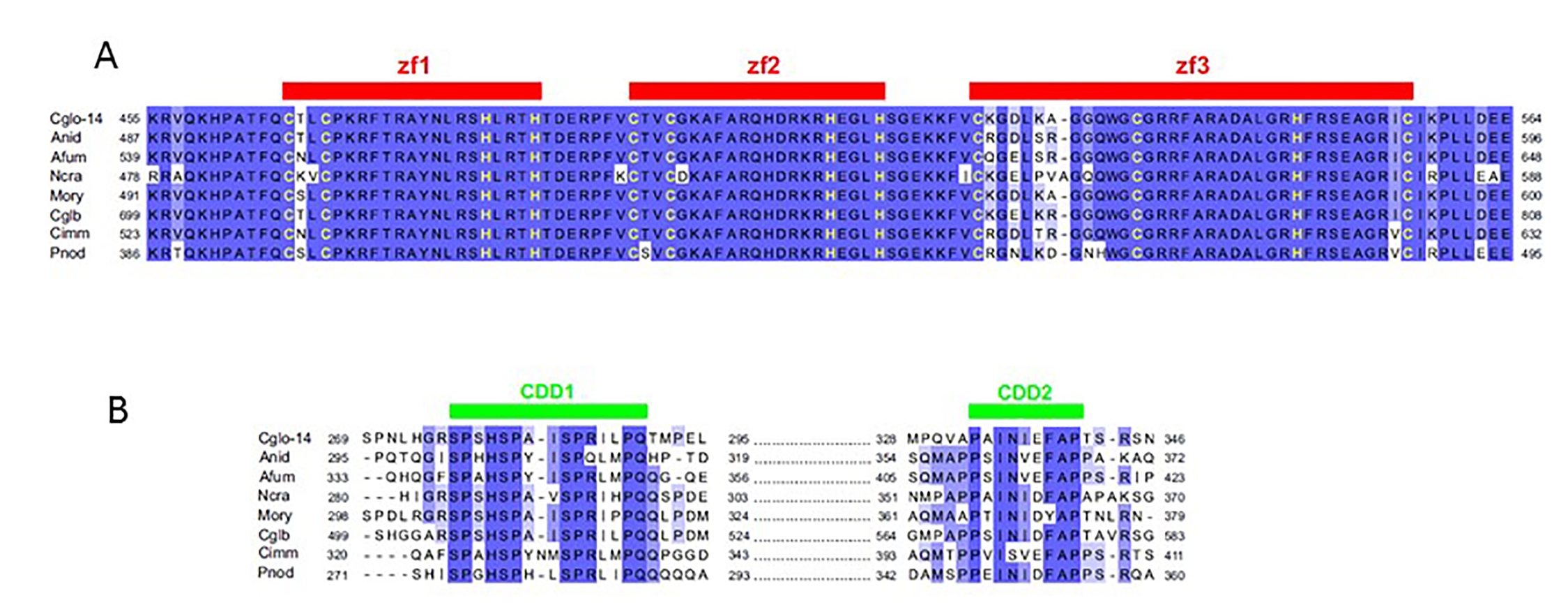

Supplement: S2 Fig — The zinc-finger region is shown (red boxes), with classical C2H2 zinc fingers zf1 and zf2, and non-canonical CCHC zinc finger zf3. Protein accession numbers are as follows: Cglo-14, C. gloesporoides EQB55543.1; Anid, A. nidulans BAE94327; Afum, A. fumigatus EAL88401; Ncra, N. crassa EAA32849; Mory, XP_359644.1 Magnaporthe oryzae; Cglb, Chaetomium globosum EAQ88414; Cimm, Coccidioides immitis EAS33001; Pnod, Parastagonospora nodorum EAT87393. Alignment of a select number of Crz1/CrzA homologs shows the putative calcineurin-docking domains (CDDs, green boxes). Protein alignments were performed using Clustal Omega online service at EBI (http://www.ebi.ac.uk/Tools/msa/clustalo/). Residue shading as in legend of S1 Fig. (TIF) [file pone.0168561.s002.tif]

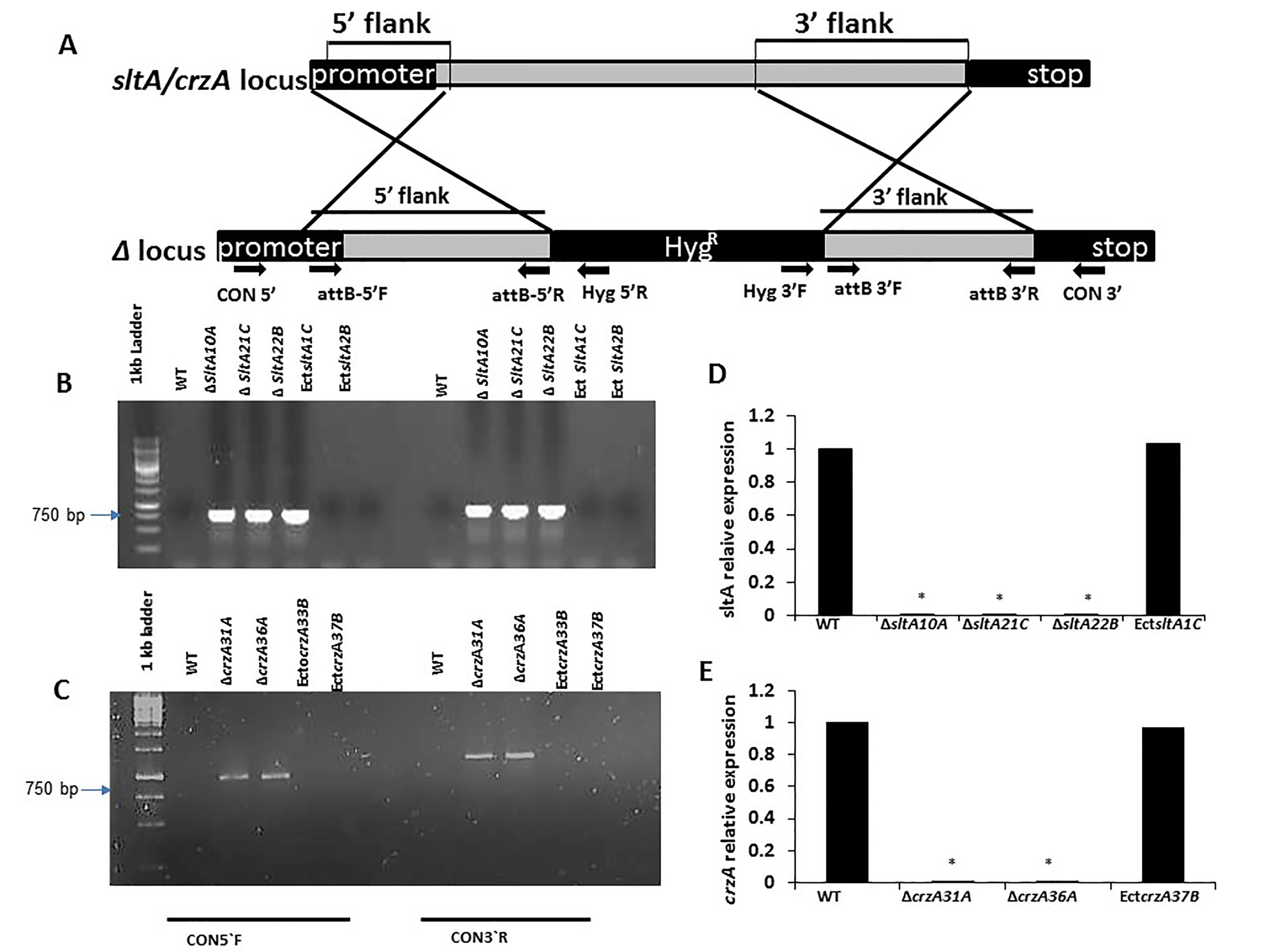

Supplement: S3 Fig — (A) Scheme describing gene disruption by homologous recombination. The pairs of primers used to create the construct were attBsltA/crzA _5'F/–attBsltA/crzA _5'R for the 5' end and attBsltA/crzA _3'F—attBsltA/crzA _3'R for the 3' end. (B) PCR analysis of the WT strain, ectopic colony (Ect), and independent sltA-disrupted colonies (ΔsltA). (C) PCR analysis of the WT strain, ectopic colony (Ect), and independent crzA-disrupted colonies (ΔcrzA). sltA/crzA _5’ctrl_F (S1 Table) flanking a position upstream of the sltA:HYG3 region and reverse primer Hyg_5'R (S1 Table) located on the hygromycin cassette were used to identify positive sltA/crzA gene replacement at the 5′ locus. Hyg_3'F (S1 Table) from the hygromycin cassette and sltA/crzA _3’ctrl_R (S1 Table) flanking the sltA:HYG3 region were used to identify sltA/crzA gene replacement at the 3′ locus. sltA/crzA attB primers for the 5' and 3' ends (S1 Table) were used for WT DNA quality control (not shown). Primers attB sltA/crzA _5'F and Hyg_5'R (S1 Table) were used as a positive control for the ectopic strains, to confirm random integration of the 5'- sltA/crzA:HYG3 cassette. (D) Relative expression of ΔsltA, WT and ectopic-integration strains, as detected by qRT-PCR. The relative expression values obtained by qRT-PCR were normalized against 18S rRNA. Values represent means ± SE of duplicates. (E) Relative expression of WT strain, ΔcrzA and ectopic-integration strains, as detected by qRT-PCR. The relative expression values obtained by qRT-PCR were normalized against 18S rRNA. Experiments were repeated three times and results of a single representative experiment are shown. Average of three technical replications is presented and asterisks marked columns are significantly different at P ≤ 0.05 according to the Student’s t-test. (TIF) [file pone.0168561.s003.tif]

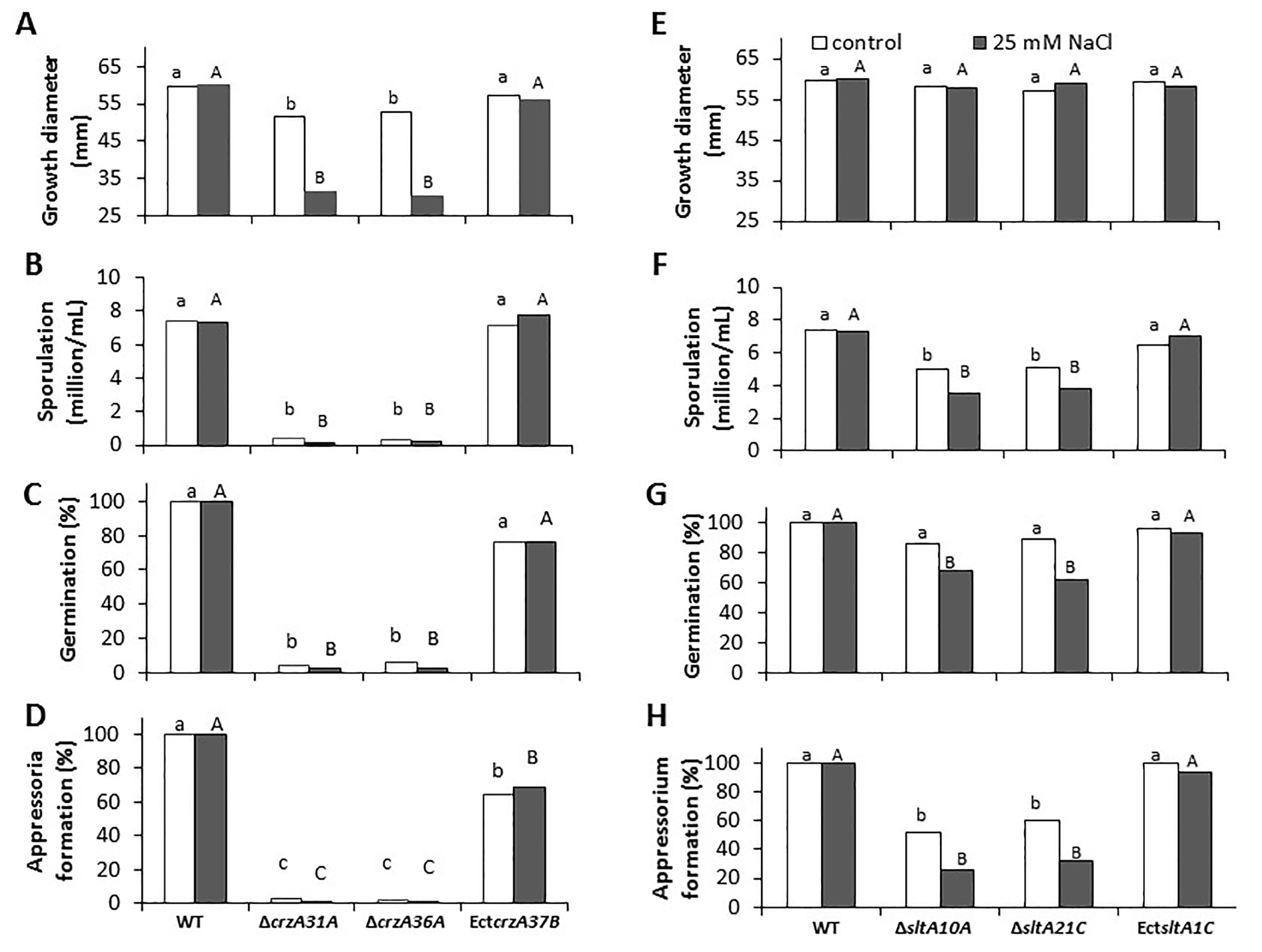

Supplement: S4 Fig — These C. gloeosporioides strains were disc-inoculated on glucose minimal media amended with 25 mM NaCl and incubated for 5 days at 24°C. (A, E) Radial colony growth, (B, F) sporulation, (C, G) germination and (D, H) appressorium formation were evaluated after 16 h of incubation on glass slides at 24°C. Experiments were repeated three times and results of a single representative experiment are shown. Columns with different letters (lower or upper case) are significantly different at P ≤ 0.05 according to the Tukey-Kramer multiple comparison test. (TIF) [file pone.0168561.s004.tif]

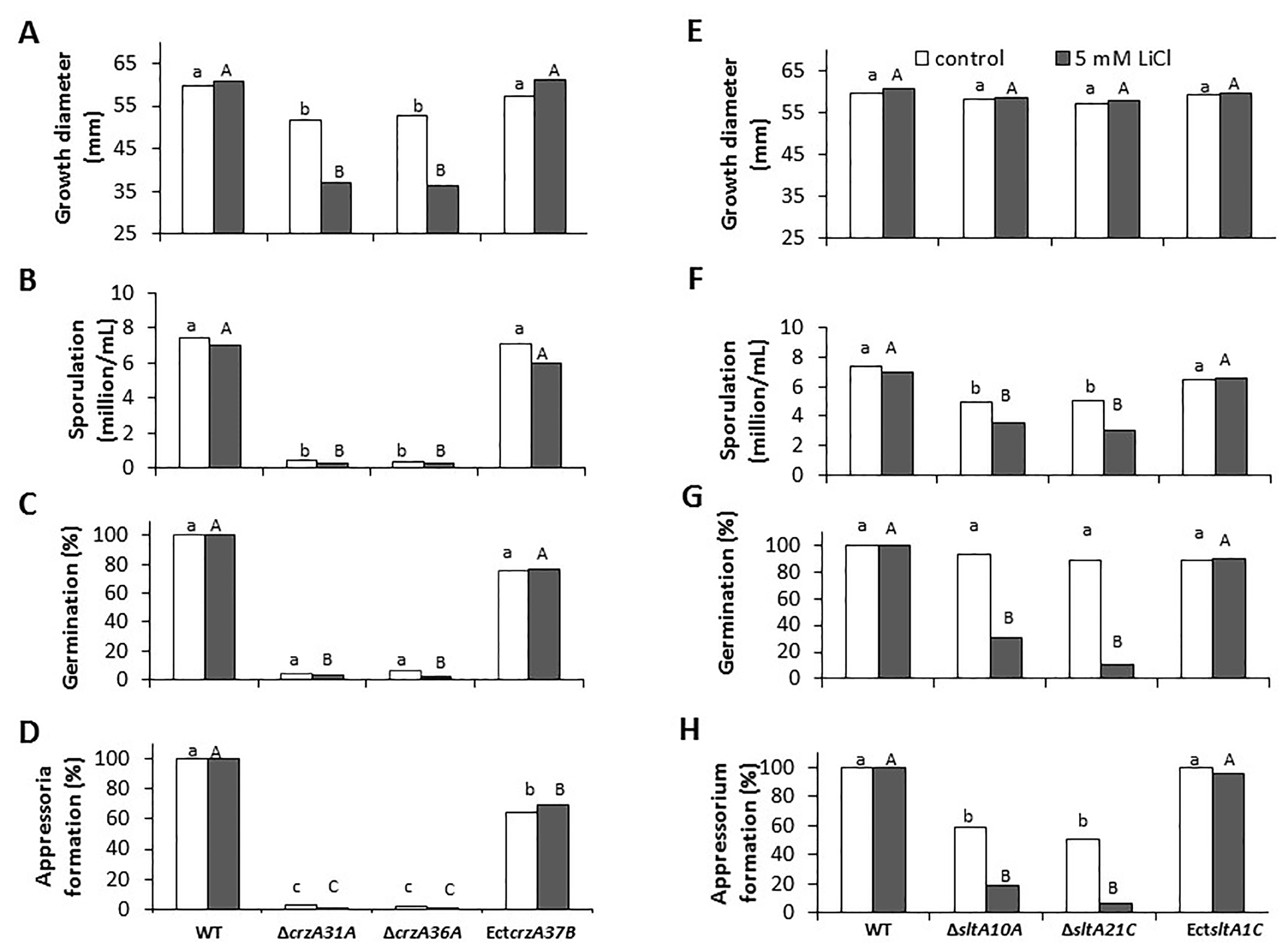

Supplement: S5 Fig — These C. gloeosporioides strains were disc-inoculated on glucose minimal media amended with 25 mM LiCl and incubated for 5 days at 24°C. (A, E) Radial colony growth, (B, F) sporulation, (C, G) germination and (D, H) appressorium formation were evaluated after 16 h of incubation on glass slides at 24°C. Experiments were repeated three times and one of the experiments is reported. Experiments were repeated three times and results of a single representative experiment are shown. Columns with different letters (lower or upper case) are significantly different at P ≤ 0.05 according to the Tukey-Kramer multiple comparison test. (TIF) [file pone.0168561.s005.tif]

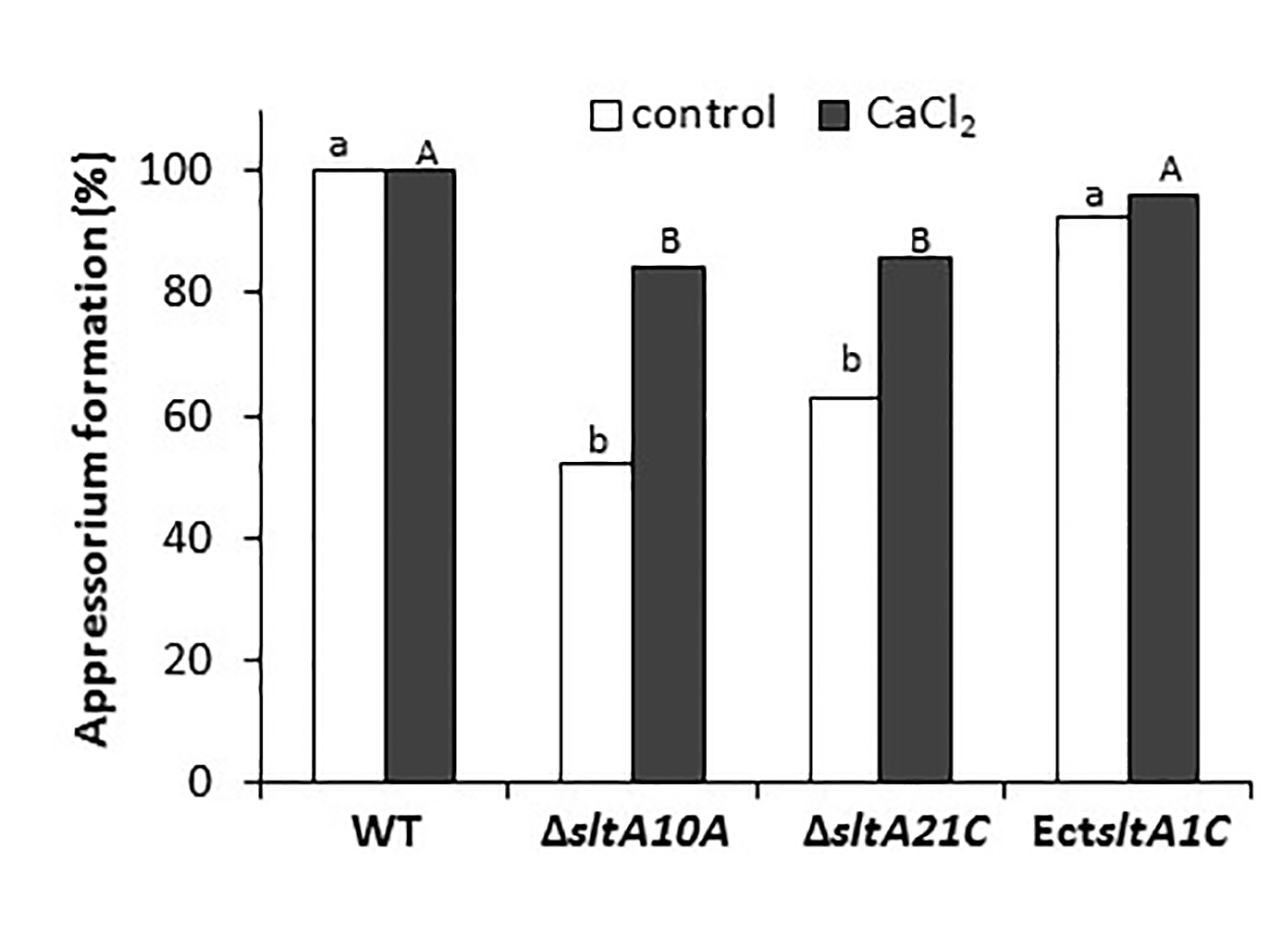

Supplement: S6 Fig — Five drops of spores of the different strains were placed on the peel of mango fruit cv. Shely and incubated in a humid plastic container overnight at 24°C. Microscopic evaluation of germination and appressorium formation was evaluated after the 0.3–0.5 mm thick peel containing the inoculated drops was excised from the fruit 24 h after inoculation. Experiments were repeated three times and results of a single representative experiment are shown. Columns with different letters (lower or upper case) are significantly different at P ≤ 0.05 according to the Tukey-Kramer multiple comparison test. (TIF) [file pone.0168561.s006.tif]

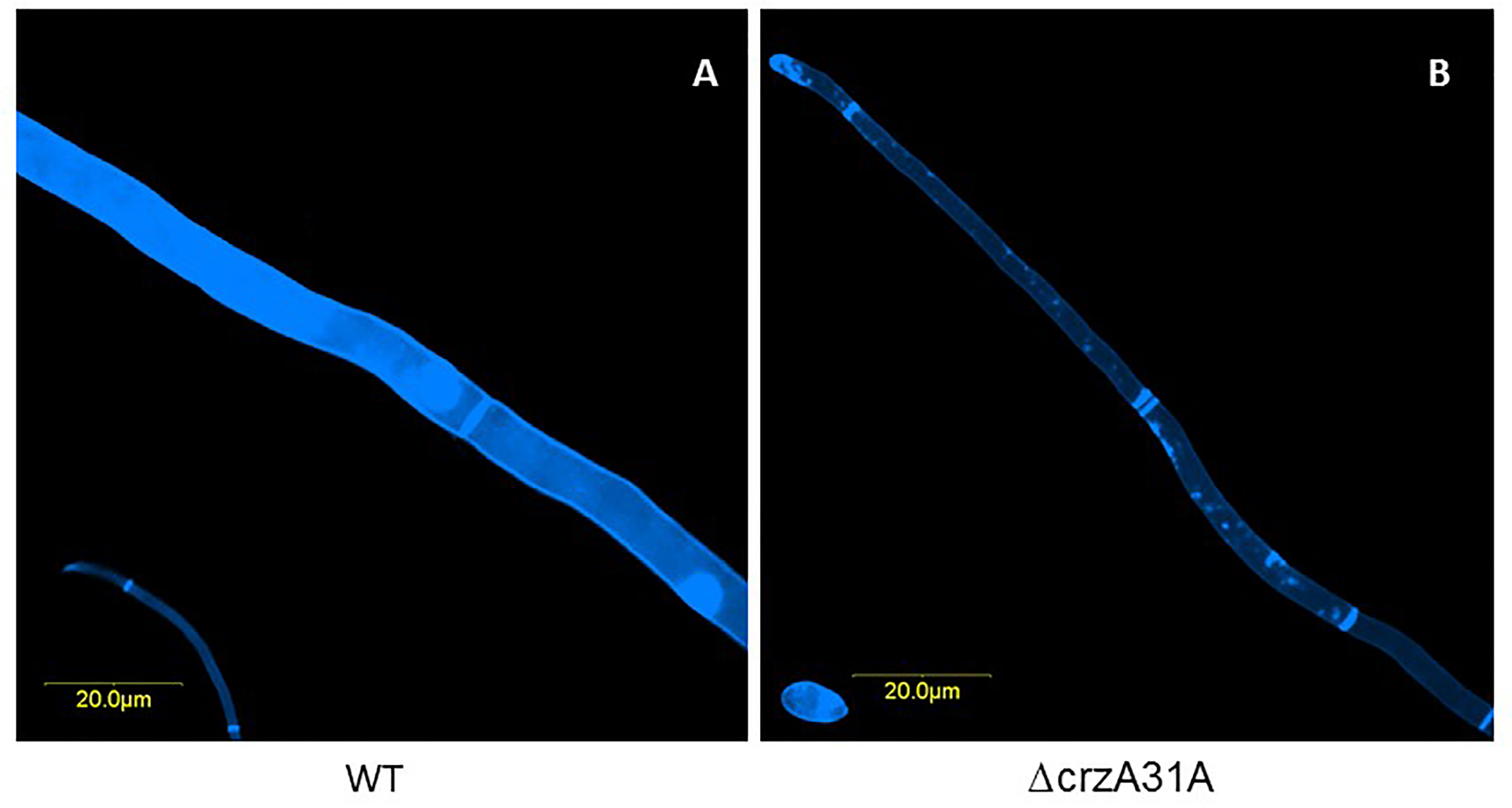

Supplement: S7 Fig — Microscopic evaluation indicate the reduced staining of the ΔcrzA31a mutant because of the downregulation of chitin synthesis. (TIF) [file pone.0168561.s007.tif]
