# Supplementary material for: Cation-Stress-Responsive Transcription Factors SltA and CrzA Regulate Morphogenetic Processes and Pathogenicity of Colletotrichum gloeosporioides
Source: PLoS One. 2016 Dec 28;11(12):e0168561. doi: 10.1371/journal.pone.0168561 (PMC5193415; doi:10.1371/journal.pone.0168561)
Supplement: S1 Table — (DOCX) [file pone.0168561.s008.docx]

| Primer name | Sequence (5'→3') |
| --- | --- |
| Hyg925 | GCCTGGACGACTAAACCAAA |
| Hyg 3 end 2 | ACCTACTACTGGGCTGCTTC |
| AttbsltA5`F | GGGGACAACTTTGTATAGAAAAGTTGGCGGCCGCTGGCCAATCCTAAAACCTTG |
| AttbsltA3`R | GGGGACAGCTTTCTTGTACAAAGTGGTCAGTTGTTTCCGTCCAACA |
| AttbcrzA5`F | GGGGACAACTTTGTATAGAAAAGTTGGCGGCCGCTTACTGCGACACGCATTCTC |
| AttbcrzA3`R | GGGGACAGCTTTCTTGTACAAAGTGGGATACCAGCGGCCAGTACA |
| sltA5 end control | CTCCTGCGTCCTCAGCTTC |
| crzA5end control | TCTGAGTCCTCCAGCTGTCC |
| sltA3 end control | GTCATACGCCTCCACCCTTA |
| crzA3 end control | AGGATAGATGCCGAGGGAGT |
| Primers used in real-time PCR | |
| SltA F | TCCTCCCATGTAGGAGTGCTT |
| SltA R | TCTCCATGTGCTGCTTGCA |
| CrzA F | GGCATTTCCGTTCTGAAGCA |
| CrzA R | CCAAGCACGTTGTCTGTCAAG |
| chsA F | ACCGCTTCCGTGCGATTA |
| chsA R | AGAGTGTGGTCACCATGGAAGTAC |
| chsB F | AGTTCCACAAGATGCGCTACAC |
| chsB R | GGGACGCAGATCGTAACCAT |
| fsk1A F | CCAAGGAGGAGGTTGAAGACAT |
| fsk1A R | CGCATACTGTCACGCTGGAA |
| Vcx1 F | CGAACAGATCTCCGTGAAGCT |
| Vcx1 R | CAATCAATTCGACGGCGTTT |
| Ena1 F | GACGCTTCCTTGGAGTGAATG |
| Ena1 R | AGCAGTGCCGAAAAGCAAGA |
| pmk1A F | GCCATCAAAAAGGTCACAAA |
| pmk1A R | TGCTGGAGCAGCTTAATCTC |
| CAT F | ATGCCATGGCGGATCCTA |
| CAT R | GTACTCGAGGTGGGCATCGAT |
| Cg18SF | `GTGAGGCCCTCAAAAGGTA |
| Cg18SR | GGATCCCAGTGCGGACG |
